# CGRP restrains CD4^+^ T cell responses and allergic sensitization

**DOI:** 10.3389/fimmu.2025.1671269

**Published:** 2025-11-18

**Authors:** Xiaoshi Li, Ying Zhang, Wenlong Chen, Qingren Meng, Ai Huang, Jiayi Pan, Duan Chen, Yue Xiao, Jialin Wei, Heng Sun, Quan Liu

**Affiliations:** 1 Department of Biochemistry, SUSTech Homeostatic Medicine Institute, University Laboratory of Metabolism and Health of Guangdong, Joint Laboratory of Guangdong-Hong Kong Universities for Vascular Homeostasis and Diseases, School of Medicine, Southern University of Science and Technology, Shenzhen, Guangdong, China; 2 Shenzhen University International Cancer Center, Guangdong Key Laboratory of Genome Instability and Human Disease Prevention, Marshall Laboratory of Biomedical Engineering, Department of Biochemistry and Molecular Biology, Shenzhen University Medical School, Shenzhen, Guangdong, China; 3 Marshall Laboratory of Biomedical Engineering, Department of Biochemistry and Molecular Biology, Shenzhen University Medical School, Shenzhen, Guangdong, China; 4 Department of Oncology, National Cancer Center/National Clinical Research Center for Cancer/Cancer Hospital & Shenzhen Hospital, Chinese Academy of Medical Sciences and Peking Union Medical College, Shenzhen, Guangdong, China

**Keywords:** CGRP, CD4^+^ T cells, asthma, allergic sensitization, neuroimmune

## Abstract

**Background:**

Calcitonin gene-related peptide (CGRP), a neuropeptide released by sensory neurons, plays an emerging role in immune regulation, yet its function in adaptive immunity remains poorly understood. Here, we identify the CGRP–RAMP1 pathway as a key intrinsic regulator of CD4^+^ T cell responses during allergic sensitization.

**Methods:**

House dust mite (HDM) was used to induce allergic sensitization in mice. CGRP^+^ sensory nerve fiber distribution in mediastinal lymph nodes (medLNs) was analyzed with whole-mount imaging. RAMP1 expression on immune cells was assessed with a RAMP1-mCherry reporter by flow cytometry. TCR-seq, parabiosis, and adoptive transfer were employed to assess the biological roles of RAMP1 expression in CD4^+^ T cells. *In vitro*, CD4^+^ T cells were stimulated, differentiated, and analyzed by flow cytometry, ATAC-seq, and RNA-seq to evaluate the impact of CGRP. CD4^+^ T cell-specific RAMP1 knockout mice and CGRP treatment were used to evaluate immune cell infiltration and Tfh responses in allergic sensitization. Additionally, *Calca^−/−^, Ramp1^−/−^*, and CD4^+^ T cell-specific RAMP1 knockout mice were used from immunological studies in the HDM-induced allergic asthma model. CGRP was intraperitoneally injected to evaluate its preventive effect on asthma.

**Results:**

CGRP^+^ fibers densely innervate medLNs. In CD4^+^ T cells, RAMP1 is preferentially expressed on naïve ones. While RAMP1 does not affect thymocyte development, TCR diversity, or tissue residency, CGRP–RAMP1 signaling suppresses CD4^+^ T cell activation and differentiation. CGRP reshapes chromatin accessibility and transcriptional programs to suppress a responsive state and repress Tfh-associated gene expression of CD4^+^ T cells. Following allergen sensitization, the density of CGRP^+^ fibers in the medLNs is reduced. CD4^+^ T cell-specific RAMP1 deficiency promotes Tfh cell accumulation and B cell activation in the medLNs, exacerbating allergic sensitization. Conversely, exogenous CGRP treatment mitigates allergic sensitization in a RAMP1-dependent manner. Finally, CGRP treatment during the sensitization phase effectively alleviates allergic asthma.

**Conclusions:**

These findings suggest a neuroimmune axis in which CGRP–RAMP1 pathway restrains allergic sensitization by directly modulating the immunobiology of CD4^+^ T cells.

## Graphical Abstract

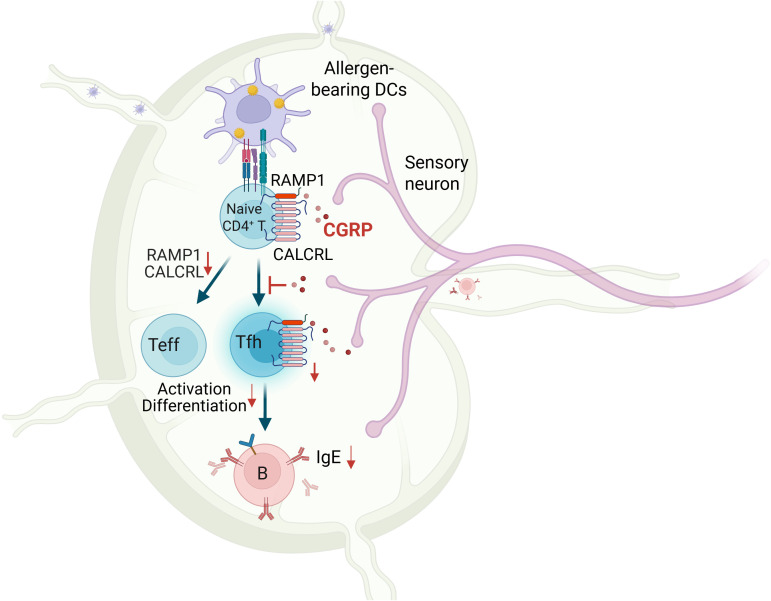

## Highlights

- CGRP^+^ sensory nerve fibers densely innervate mediastinal lymph nodes, and their density is reduced following allergen sensitization.
- CGRP–RAMP1 pathway constrains CD4^+^ T cell activation and differentiation.
- CD4^+^ T cell-specific RAMP1 deficiency promotes Tfh accumulation and allergic sensitization.
- Exogenous CGRP treatment attenuates allergic sensitization in a RAMP1-dependent manner.

## 1 Introduction

Neural and immune systems are tightly interconnected through complex bidirectional communication networks that play critical roles in maintaining tissue homeostasis and orchestrating immune responses to injury, infection, and inflammation (1–4). Sensory nerve fibers innervate hematopoietic organs, barrier tissues, and secondary lymphoid organs. Several neuropeptides, such as substance P (SP), calcitonin gene-related peptide (CGRP), and norepinephrine (NE), have been detected in lymph nodes (5, 6). In mice, Nav1.8^+^ sensory fibers densely innervate the lymph node hilus and directly interact with subcapsular macrophages and naïve lymphocytes beneath the capsule (5, 7), suggesting a functional neuroimmune unit that may locally modulate adaptive immune responses.

Allergic sensitization represents the initiating phase of allergic asthma and a prerequisite for type 2 inflammation upon allergen rechallenge (8). Allergic asthma is a chronic airway disorder characterized by eosinophilic infiltration, elevated IgE, and airway hyperresponsiveness (9), and it affects over half of pediatric and adult asthma cases (10). Despite the therapeutic efficacy of inhaled corticosteroids and biologics in most patients, those with severe or refractory asthma often experience persistent inflammation and frequent exacerbations (11). Notably, mast cells and eosinophils may remain in lung tissues and sustain type 2 cytokine production even after apparent resolution of inflammation (11). T follicular helper (Tfh) cells, which promote germinal center responses and IgE class-switching in B cells, are increasingly recognized as critical regulators of sensitization and chronicity in allergic asthma. Thus, early modulation of Tfh cell differentiation during sensitization may be a promising strategy for targeted immunotherapy.

Immunomodulatory molecules have emerged as promising therapeutic candidates and are increasingly employed in the clinical management of immune-mediated disorders. CGRP is a neuropeptide predominantly secreted by sensory neurons and pulmonary neuroendocrine cells (PNECs), and plays essential roles in immune regulation. Through binding to its canonical receptor complex composed of RAMP1 and CALCRL, CGRP inhibits ILC2 expansion and IL-13 production and mitigates IL-33-driven airway inflammation in nonallergic contexts (12). However, whether the CGRP–RAMP1 axis modulates Tfh cell response during allergen sensitization, and whether it contributes to T cell-driven pathology in allergic asthma remain unknown.

In this study, we revealed a previously unappreciated role for the CGRP–RAMP1 pathway in suppressing CD4^+^ T cell activation and restraining Tfh cell accumulation during the sensitization phase of allergic asthma. With genetic models, including a fluorescent RAMP1 reporter and T cell-specific RAMP1 knockout mouse, combined with CGRP treatment, we define the functional landscape of CGRP–RAMP1 signaling in regulating adaptive immune priming. Collectively, our findings establish CGRP–RAMP1 signaling as a key modulator of allergen sensitization and provide mechanistic insight into how sensory neurons regulate mucosal immune responses.

## 2 Materials and methods

### 2.1 Mice

C57BL/6, B6.SJL-*Ptprc^a^Pepc^b^*/BoyJ (B6.CD45.1), NOD.Cg-*PrkDC^cid^ Il2rg^tm1Wjl^*/SzJ (NSG) and B6.Cg-Tg(Cd4-cre)1Cwi/BfluJ (*Cd4^cre^*) mice were purchased from the Jackson Laboratory. B6.*Ramp1^−/−^* and B6.*Calca^−/−^* mice were purchased from Cyagen Biosciences. B6.*Ramp1-mCherry^fl/fl^* mice were generated via the gene targeting of embryonic stem (ES) cells, which were provided commercially by Cyagen Biosciences. Male mice aged 8–12 weeks were used in the experiments. All mice were kept in specific pathogen free environment under a 12-h light and dark cycle, at a constant temperature of 21 ± 1 °C, with humidity maintained at 40%–70%. Mice were provided with autoclaved food and water. All mouse experiments were approved by and performed in accordance with the Animal Care and Use Committee at the Southern University of Science and Technology.

### 2.2 Whole-mount immunohistochemistry

Whole-mount immunohistochemistry of medLNs was performed following a modified iDISCO protocol (5, 13). Briefly, mice were deeply anesthetized with 2.5% avertin (Sigma-Aldrich, USA, T48402) and perfused with PBS followed by 4% paraformaldehyde (PFA; Sigma-Aldrich, USA, 158127). MedLNs were harvested, fixed in 4% PFA overnight at 4 °C, and dehydrated through a methanol gradient. Tissues were permeabilized in PBS containing 0.3% Triton X-100, 20% DMSO, and 0.3 M glycine overnight, and then blocked with 10% DMSO and 6% donkey serum in PBS supplemented with 0.3% Triton X-100. Samples were incubated with CGRP (D5R8F; CST, USA, 14959S) Rabbit mAb in PTwH buffer (PBS with 0.3% Triton X-100, 0.3% Tween-20 and 10 mg/mL heparin) containing 5% DMSO and 3% donkey serum for 3 days, followed by Alexa Fluor 594 Donkey anti-rabbit IgG (minimal x-reactivity) Antibody (Invitrogen, USA, 406418) incubation under the same conditions. After washing, tissues were cleared in methanol and BABB (1:2 benzyl alcohol:benzyl benzoate; ansiang, China, L-AX0215), and imaged using a Zeiss LSM 980 confocal microscope. Image analysis and 3D reconstruction were performed with Imaris software (Bitplane AG, Switzerland). For quantification of penetration depth of sensory fibers, we first reconstructed a whole-node surface using the CGRP^+^ channel to define the region of interest. The surface was inspected in orthogonal views and minimally edited to match the capsule boundary. With this surface selected, we ran Tools Distance Transformation and chose inside. This generated a new channel in which each voxel intensity equals the shortest Euclidean distance (in µm) from that voxel to the outside of the surface; voxels outside the surface are set to 0. Thus, higher values indicate greater depth from the capsule toward the node interior. To assess nerve localization, we analyzed the CGRP channel with respect to this distance map. Specifically, we computed Intensity Max (and related statistics) for CGRP within distance-defined shells to compare peripheral (near-capsule) versus interior signal.

### 2.3 Immunofluorescence

Lungs, medLNs and spleens were fixed in 2% PFA, cryoprotected in 30% sucrose, embedded in optimal cutting temperature (OCT), and cryosectioned at a thickness of 6 μm thickness. The sections were permeabilized with 0.1% Triton X-100, blocked in 2% BSA, and stained with mCherry Monoclonal Antibody (16D7) Alexa Fluor™ 594 (Thermo Scientific, USA, M11240). Nuclei were counterstained with DAPI.

### 2.4 Tissue collection and single-cell suspension

Mouse BALF was collected by flushing the trachea twice with 1 mL of PBS as previously described (14). Lungs were minced and digested with 1 mg/mL collagenase IV (Thermo Scientific, USA, C5138) and 200 U/mL DNase I (Thermo Scientific, USA, DN25) at 37 °C for 30 min, filtered through a 100-μm mesh, and fractionated with a 40%/80% Percoll (GE, USA, 17-0891-09) gradient. Bone marrow, thymus, spleen, and medLNs were dissociated through a 70-μm strainer. Red blood cells were lysed with ammonium-chloride-potassium lysis buffer (ACK) buffer. Bone marrow cells were flushed from femurs and tibias. Cell suspensions were washed, counted, and used for further analyses.

### 2.5 Flow cytometry

Cells were preincubated with αCD16/32 (BioLegend, USA, 101302) and stained with a Zombie NIR Fixable Viability Kit (BioLegend, USA, 423106). Surface staining was performed at 4 °C for 30 min using fluorochrome-conjugated antibodies (**Supplementary Table S1**). For intracellular marker detection, the cells were fixed and permeabilized with the Foxp3/Transcription Factor Staining Buffer Set (eBioscience, USA, 00-5523-00), followed by antibody staining for 50 min at room temperature. Data were acquired on a BD FACSAria III and analyzed with FlowJo v10.8 (BD Biosciences, USA).

### 2.6 RT–qPCR

CD4^+^ T cells were isolated from spleens or medLNs via the MojoSort Mouse CD4 T Cell Isolation Kit (Biolegend, USA, 480033). Total RNA was extracted via the FastPure Cell/Tissue Total RNA Isolation Kit V2 (Vazyme, China, RC112-01), reverse transcribed into cDNA, and analyzed with TB Green Premix Ex Taq (Takara, JPN, RR820B). Gene expression was calculated using the ΔCt method. Primer sequences are listed in **Supplementary Table S2**.

### 2.7 Western blot

CD4^+^ T cells were isolated from spleens via the MojoSort Mouse CD4 T Cell Isolation Kit. Total protein was extracted from cells and then analyzed using SDS–PAGE. The target protein was transferred to PVDF membranes (Millipore, Germany, IPVH00010) for primary antibody incubation. Rabbit antibodies against extracellular RAMP1 (Alomone labs, IL, #ARR-021) or β-actin (Proteintech, China, 10094-1-AP) were used. Protein bands were visualized using the horseradish peroxidase western blot detection kit (Tannon, China, 180-5001).

### 2.8 TCR sequencing

RAMP1^+^ and RAMP1^−^ CD4^+^ T cells were sorted from *Ramp1-mCherry^fl/fl^* splenocytes by flow cytometry. DNA (∼3 μg per sample from 3.6 × 10^6^ cells) was extracted for TCR sequencing, which was performed by Jiangxi HaploX Biotechnology (China) via the Illumina NextSeq500 or HiSeq X10 platform (2×150 bp).

### 2.9 Parabiosis

CD45.1 and *Ramp1-mCherry^fl/fl^* CD45.2 mice were surgically joined as previously described (15, 16). In brief, mice were co-housed for 2 weeks prior to surgery. Skin incisions were made from elbow to knee and joints sutured with absorbable stitches. Postoperatively, mice received saline, carprofen, and 10 days of oral sulfamethoxazole (2 mg/mL; Sigma, USA, S7507-10G) and trimethoprim (0.4 mg/mL; Sigma, USA, 92131-1G). Tissues were harvested 8 weeks post-surgery for flow cytometry analysis.

### 2.10 Adoptive transfer of CD4^+^ T cells

CD4^+^ T cells were isolated from *Ramp1-mCherry^fl/fl^* spleens using the MojoSort Mouse CD4 T Cell Isolation Kit and sorted into Zombie^−^CD4^+^CD8^−^RAMP1^+^ and RAMP1^−^ subsets. Each NSG mouse received 2 × 10^5^ sorted cells via tail vein injection. Tissues were analyzed at 4 weeks post-transfer via flow cytometry.

### 2.11 Synthetic CGRP

Rat CGRP peptide (GenScript, China, RP11095) is a synthetic product with the amino acid sequence of SCNTATCVTHRLAGLLSRSGGVVKDNFVPTNVGSEAF. Working solutions were prepared in sterile PBS and adjusted to the indicated concentrations for *in vitro* stimulation (100 nM) or *in vivo* administration (5 µg in 50 μL/mouse).

### 2.12 CD4^+^ T cell isolation, culture, and stimulation

CD4^+^ T cells were isolated from spleens via the MojoSort Mouse CD4 T Cell Isolation Kit, labeled with Celltrace Blue Cell Proliferation Kit (Thermo Scientific, USA, C34568). Splenic CD4^+^ T cells seeded at 2 × 10^5^ cells/well into 96-well plates pre-coated with αCD3 (1 μg/mL; ebioscience, USA, 14-0032-85) and αCD28 (1 μg/mL; ebioscience, USA,14-0281-86). Cells were treated daily with 100 nM CGRP or PBS. After 72 h, supernatants were analyzed using Cytometric Bead Array (BD, USA, 558266&560485), and cells were subjected to flow cytometry. For IL-2 supplementation, cultures received CGRP alone or in combination with recombinant IL-2 (0, 25, 50, or 100 U/mL; PeproTech, USA, 2112-12-20).

Th cell polarization. CD4^+^ T cells were stimulated under the following conditions: Th1: 1 μg/mL αCD3, 1 μg/mL αCD28, 10 ng/mL IL-12 (PeproTech, USA, 200-12H), 1 μg/mL αIL-4 (Biolegend, USA, 500838); Th2: 1 μg/mL αCD3, 1 μg/mL αCD28, 10 ng/mL rIL-4 (PeproTech, USA, 214-14-50), 5 μg/mL αIFN-γ (Biolegend, USA, 505834); Th17: 2 μg/mL αCD3, 1 μg/mL αCD28 + 20 ng/mL IL-6 (PeproTech, USA, 200-06-20UG), 2 ng/mL TGF-β (PeproTech, USA, 100-21-10UG), αIFN-γ (Biolegend, USA, 505834), 1 μg/mL IL-4. Then, CGRP (100 nM) or PBS was added from the beginning of culture. After 72 h, cells were analyzed by flow cytometry.

### 2.13 RNA-seq

1 × 10^6^ splenic CD4^+^ T cells were stimulated with αCD3 and αCD28 in the presence of PBS or 100 nM CGRP for 24 h. Total RNA was extracted, and libraries were prepared using poly(A) selection and reverse transcription. Sequencing was performed on an Illumina HiSeq X10 or NextSeq500 platform (HaploX Biotechnology). Differentially expressed genes were defined as p < 0.05, |log_2_FC| > 0.85, reads > 300.

### 2.14 ATAC-seq

9 × 10^4^ splenic CD4^+^ T cells activated with αCD3 and αCD28, and treated with PBS or 100 nM CGRP were subjected to ATAC-seq using the Active Motif ATAC-Seq kit (Active Motif, USA, 53150). Sequencing was performed on an Illumina NovaSeq 6000 platform (HaploX Biotechnology). Reads were trimmed (Fastp), aligned to mm10 (Hisat2), and filtered (SAMtools, Picard). Peaks were called using MACS2, and data were visualized using DeepTools and the WashU Epigenome Browser.

### 2.15 BMDC isolation, culture and stimulation

Murine BMDCs were generated as previously described (17). Briefly, bone marrow cells were cultured in complete medium (RPMI 1640 containing 10% FBS, 100 U/mL penicillin, 100 μg/mL streptomycin, 2 mM L-glutamine, 1 mM sodium pyruvate, 1× MEM non-essential amino acids, 50 μM 2-mercaptoethanol, and 10 mM HEPES) supplemented with 20 ng/mL GM-CSF (PeproTech, USA, 300-03) and 10 ng/mL IL-4 for 6 days. For proliferation assays, 100 nM CGRP was added from day 0. For antigen uptake, day-6 BMDCs were incubated with DQ-OVA (1 or 10 μg/mL) ± CGRP at 37 °C or 4 °C for 2 h. For activation, day-6 BMDCs were treated with 100 nM CGRP ± 125 μg/mL Poly(I:C) (APExBIO, USA, B5551), 200 ng/mL IFN-γ (PeproTech, USA, 315-05), or 300 ng/mL GM-CSF for 24 h, followed by flow cytometry analysis.

### 2.16 Induction of allergic sensitization and allergic asthma

Allergic sensitization was performed as previously described (18, 19). On day 0, mice were anesthetized with 2.5% avertin and intratracheally instilled with 100 μg of HDM in 50 μL PBS. For CGRP treatment, mice received intraperitoneal injections of CGRP (5 μg per mouse, twice daily) from day 0 to day 6. On day 7, medLNs and BALF were collected for further analysis.

Allergic asthma was established following a sensitization and challenge protocol (19). In the sensitization phase, mice were brief anesthetized with isoflurane (RWD Life Science, China, R510-22-10) and intranasally instilled 0.1 μg HDM in 40 μL PBS on day 0. From days 7 to 11, mice were challenged daily with 10 μg HDM in 40 μL PBS. CGRP (5 μg per mouse, twice daily) was administered intraperitoneally from day 0 to day 6. On day 15, BALF and lung tissues were harvested for further analysis.

### 2.17 Hematoxylin-eosin and periodic acid-Schiff staining

Lungs were fixed in 4% PFA overnight and embedded in paraffin. Tissue sections (6 μm) were stained with H&E or PAS, and scanned using a Leica pathology scanner (Leica, USA, Aperio VERSA 8). Quantitative image analysis was performed in ImageJ. Inflammatory regions in H&E-stained sections were quantified using the Trainable Weka Segmentation plugin (20, 21), while PAS^+^ mucus-producing areas were quantified by color deconvolution (22). Results were expressed as the percentage of stained area relative to total lung tissue.

### 2.18 Statistical analysis

GraphPad Prism was used for statistical analyses. Results were expressed as mean ± SD (unless otherwise specified). The ROUT method was utilized for outlier identification, followed by the removal of approximately 10% of the most probable outliers from the dataset. A comparison between two groups was performed using a two-sided Student’s *t* test or Student’s *t* test with Holm-Sidak correction for multiple comparison for unpaired samples. For multiple comparisons on a single data set, a one-way ANOVA was performed, followed by Tukey’s multiple comparison test. Statistical significance was set at p < 0.05. Sample sizes (biological replicates = mice in each group, unless otherwise specified) show statistical significance in similar group sizes with normal variation and similar variance between groups. No randomization was used as all mice were genetically defined and inbred.

## 3 Results

### 3.1 CGRP^+^ fibers are enriched in medLNs, and RAMP1 is expressed by CD4^+^ T cells

**Figure 1:**
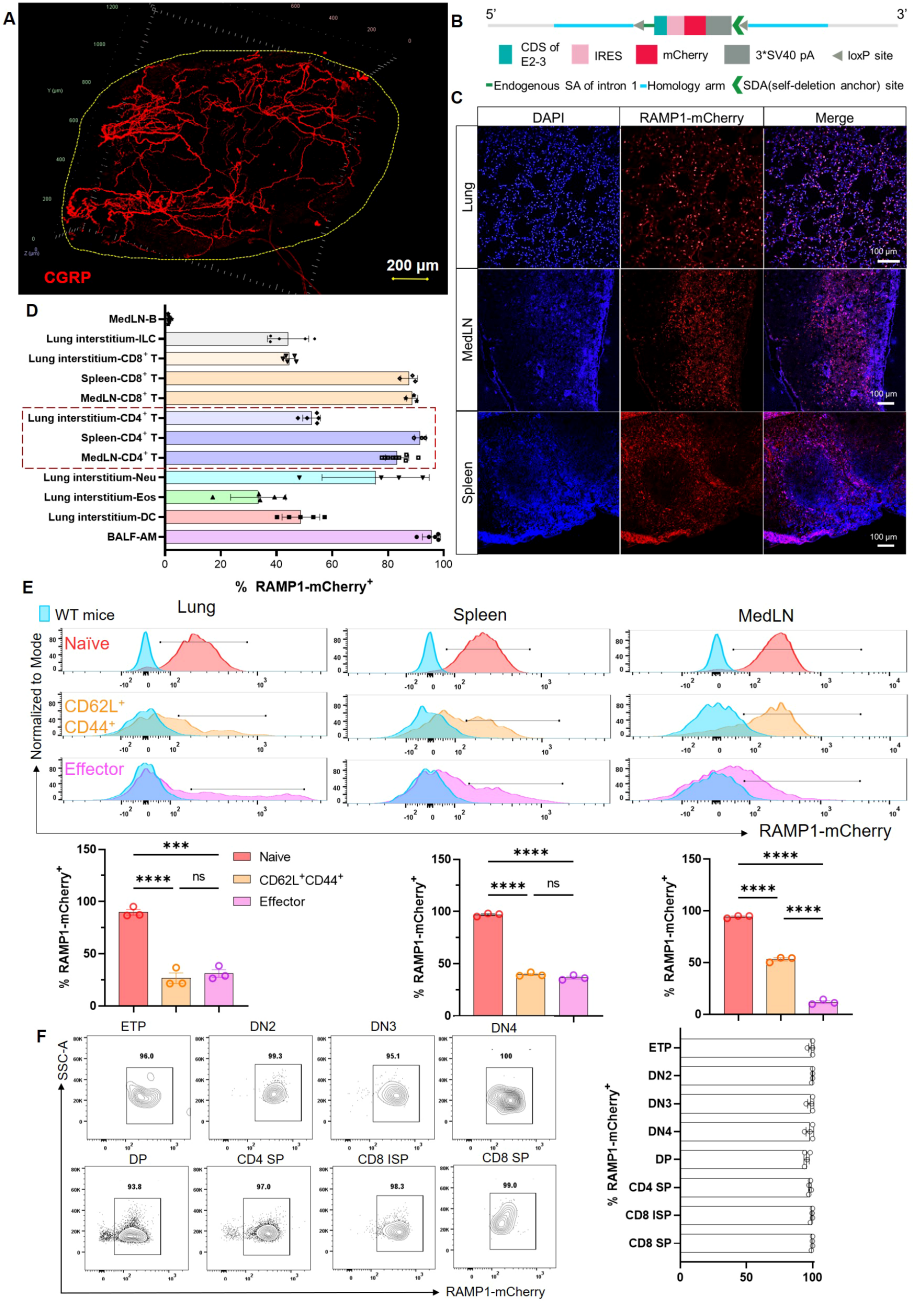
Spatial distribution of CGRP^+^ nerve fibers and RAMP1^+^ immune cells in lymphoid organs. **(A)** Whole-mount immunohistochemistry image showed CGRP^+^ nerve fibers (red) distributed in the medLN. **(B)** Schematic representation of the *Ramp1-mCherry*^fl/fl^ reporter allele design, enabling cell-specific RAMP1 expression tracking via mCherry fluorescence. **(C)** Immunofluorescence staining of lung, medLN, and spleen from *Ramp1-mCherr*y^fl/fl^ mice showing RAMP1-mCherry^+^ cells (red), DAPI nuclear counterstaining (blue), and merged images. **(D)** Flow cytometric percentages of RAMP1 expression RAMP1-mCherry^+^ in immune cell subsets, including B cells (Zombie^–^TCRβ^–^CD19^+^), ILCs (Zombie^–^lineage^–^CD45^+^CD90.2^+^), CD8^+^ T cells (Zombie^–^B220^–^CD45^+^CD11b^–^CD11c^–^TCRβ^+^CD4^–^), CD4^+^ T cells (Zombie^–^B220^–^CD45^+^CD11b^–^CD11c^–^TCRβ^+^CD4^+^), neutrophils (Neu, Zombie^–^B220^–^CD45^+^ CD11b^+^CD11c^–^Siglec-F^–^Ly6G^+^), eosinophils (Eos, Zombie^–^B220^–^CD45^+^CD11b^+^CD11c^–^Siglec-F^+^Ly6G^–^), DCs (Zombie^–^B220^–^CD45^+^CD11b^+^ CD11c^+^Siglec-F^–^Auto^–^), and AMφ (Zombie^–^B220^–^CD45^+^CD11b^lo^CD11c^+^Siglec-F^+^Auto^+^) in medLNs, lung interstitum, spleens or BALF, n = 3–9/group; **(E)** Representative histograms and bar graphs showed the percentages of RAMP1-mCherry^+^ in naïve (CD62L^+^CD44^−^, red), central memory (CD62L^+^CD44^+^, orange), and effector (CD62L^−^CD44^+^, pink) CD4^+^ T cells from lungs, spleens, and medLNs of *Ramp1-mCherry^fl/fl^* and WT mice (blue), n = 3/group. **(F)** Flow cytometry contour plots and bar graphs showed the percentages of RAMP1-mCherry^+^ in early thymocyte progenitor (ETP), double-negative cell (DN) 2–4, double-positive cell (DP), CD4^+^ single-positive cell (SP), CD8^+^ immature SP, and CD8^+^ SP, n = 4/group. Graphs depict individual values and group means ± SD. Statistical significance was determined using one-way ANOVA with Tukey’s multiple comparison test **(E)**: ns, not significant; ***P < 0.001; ****P < 0.0001.

To examine the spatial distribution of CGRP within lymphoid tissues, we used a protocol adapted from the immunolabeling-enabled 3D imaging of solvent-cleared organs (iDISCO) method (5, 13). This approach enabled high-resolution volumetric imaging, revealing that CGRP^+^ nerve fibers were densely distributed along the mediastinal lymph nodes (medLNs) (**Figure 1A**). To track immune cell-specific expression of RAMP1, we generated *Ramp1-mCherry^fl/fl^* reporter mice by inserting a loxP–IRES–mCherry cassette into intron 1 of the *Ramp1* locus (**Figure 1B**). Immunofluorescence staining of the lung, spleen, and lymph nodes demonstrated robust mCherry signal, indicating widespread expression of RAMP1^+^ cells in mucosal and lymphoid compartments (**Figure 1C**). Flow cytometric profiling revealed high RAMP1 expression in alveolar macrophages (AMφ), CD4^+^ and CD8^+^ T cells, innate lymphoid cells (ILCs), dendritic cells (DCs), eosinophils, and neutrophils, but minimal expression in B cells, with the strongest signals observed in AMφ and T cells (**Figure 1D**). Given that CD4^+^ T cells constitute a major immune subset in the lung, medLN and spleen, we further examined RAMP1 expression across CD4^+^ T cell subsets. Naïve cells (CD62L^+^CD44^−^) showed the strongest expression, which was decreased in the central memory and effector populations (**Figure 1E**). In line with these findings, RAMP1 was broadly expressed across T cell developmental stages, including early thymocyte progenitor (ETP), double negative 2 (DN2)–DN4, and single positive (SP) cells (**Figure 1F**). These data suggest that CGRP–RAMP1 may play a previously underappreciated role in the early activation and lineage commitment of CD4^+^ T cells within sensory nerve-rich lymphoid niches.

### 3.2 RAMP1 expression does not affect thymocyte development, TCR diversity or tissue residency of CD4^+^ T cells

RAMP1 was highly expressed across multiple stages of thymocyte development, raising the question of whether it influences T cell output or peripheral composition. To validate our model, we confirmed efficient *Ramp1* deletion in CD4^+^ T cells from *Cd4^cre^Ramp1-mCherry^fl/fl^* (CD4^+^ T cell-specific *Ramp1*-deficient, CD4^Δ^*^Ramp1^*, **Supplementary Figure S1**). However, quantification of total thymocytes, including ETP, DN2–DN4, DP, and SP subsets, revealed no significant differences between CD4^Δ^*^Ramp1^*, *Ramp1^−/−^*, *Calca^−/−^* mice, and their wild-type (WT) controls (**Supplementary Figure S2A**). Similarly, splenocyte numbers, as well as mature CD4^+^ and CD8^+^ T cell frequencies in the spleens, remained unaltered (**Supplementary Figure S2B**), indicating that neither global CGRP deficiency nor T cell-intrinsic loss of RAMP1 impacts thymic development or peripheral T cell seeding. Given the high expression of RAMP1 on CD4^+^ T cells and its proposed role in modulating T cell responsiveness, we next evaluated whether RAMP1 expression affects the diversity of the T cell receptor (TCR) repertoire, a critical indicator of adaptive immune potential (23). TCR sequencing analysis of RAMP1-mCherry^+^ and RAMP1-mCherry^−^ CD4^+^ T cells from the spleens revealed no significant differences in V–J usage, clonotype number, CDR3 length distribution, or the Shannon diversity index (**Supplementary Figures S2C, D**). Although a trend toward increased diversity was observed in RAMP1-mCherry^+^ T cells, this trend did not reach statistical significance.

Recent studies have shown that certain lymphocyte subsets, such as ILC2s and memory T cells, exhibit long-term residency within specific tissues (24–26). To evaluate whether RAMP1 expression is associated with this residency phenotype, we employed a classical parabiosis model (27) by surgically conjoining CD45.2 *Ramp1-mCherry^fl/fl^* donor mice with CD45.1 wild-type recipients (**Supplementary Figure S3A**). After 8 weeks of shared circulation, donor-derived T cells, eosinophils and neutrophils were detected in both parabionts, whereas ST2^+^ ILC2s, mast cells and AMφ remained largely confined to the donor (**Supplementary Figure S3B**). Both the circulating and resident immune populations, including the above subsets, expressed RAMP1, suggesting that RAMP1 expression does not inherently define migratory behavior (**Supplementary Figures S3C−G**). Donor-derived CD4^+^ T cells efficiently trafficked to the BM, spleen, medLN, and lung, with minimal reentry into the thymus. RAMP1 expression was comparable between donor- and recipient-derived CD4^+^ T cells across the tissues examines, suggesting that RAMP1 expression has no impact on migration or retention (**Supplementary Figure S3D**). Similar expression profiles were observed in CD8^+^ T cells, eosinophils, and neutrophils (**Supplementary Figure S3E–G**). These findings suggesting that RAMP1 is not associated with tissue retention and does not affect leukocyte residency.

To explore potential plasticity in RAMP1 expression, we performed adoptive transfer of FACS-sorted RAMP1-mCherry^+^ and RAMP1-mCherry^−^ CD4^+^ T cells into NOD-*scid* IL2Rg^null^ (NSG) mice (**Supplementary Figure S4A**). After 4 weeks, both populations expanded and populated immune tissues. Interestingly, RAMP1 expression among recovered CD4^+^ T cells was similar between the groups (**Supplementary Figure S4B**), suggesting that *in vivo* phenotypic plasticity between RAMP1^+^ and RAMP1^−^ CD4^+^ T cells, is potentially influenced by as-yet undefined cues during homeostatic expansion or tissue adaptation.

### 3.3 CGRP restrains CD4^+^ T cell activation and effector differentiation

**Figure 2:**
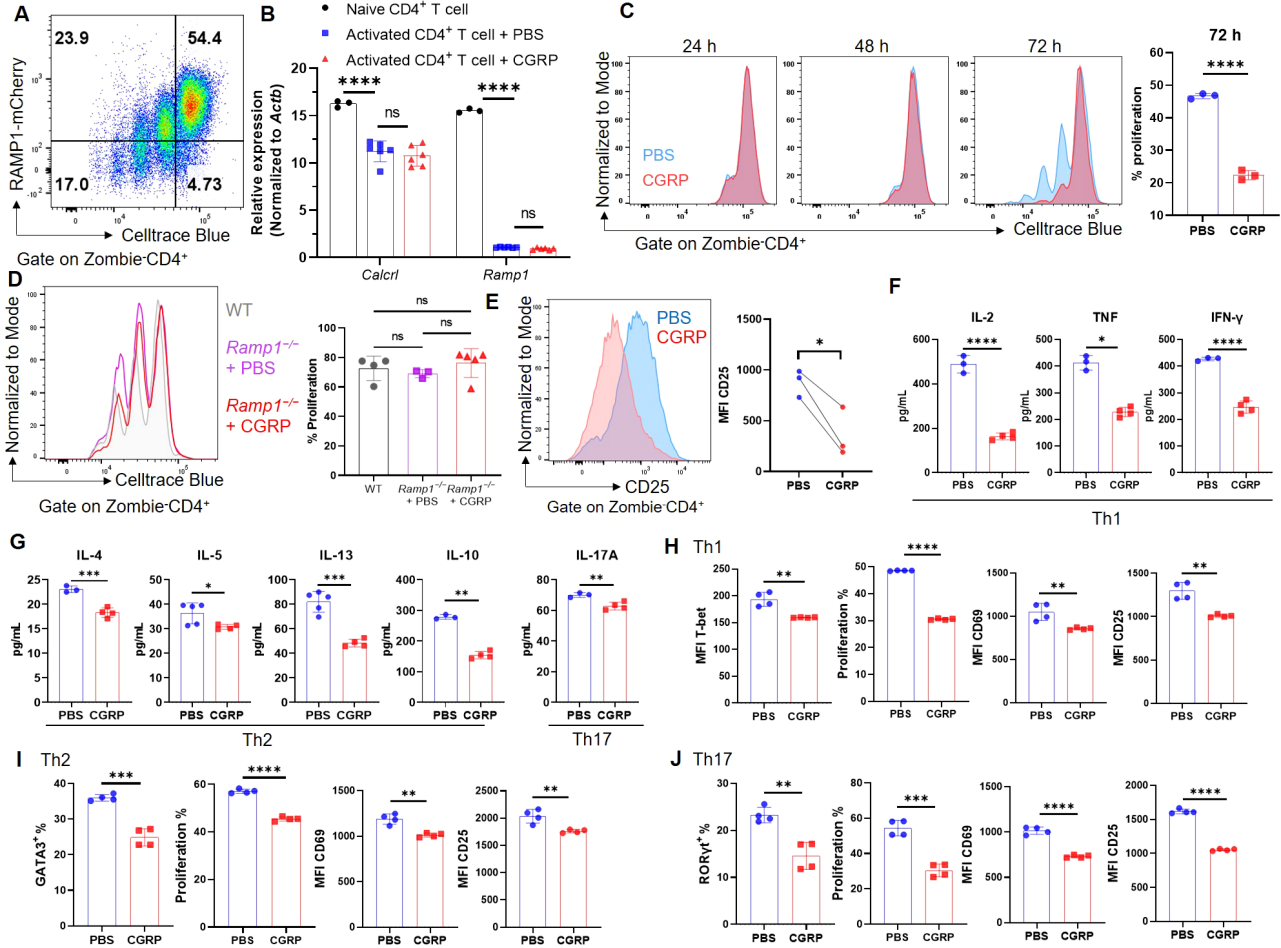
CGRP suppresses CD4^+^ T cell activation and differentiation. **(A)** Representative flow cytometry plot showed RAMP1-mCherry expression in activated CD4^+^ T cells upon αCD3 and αCD28 stimulation, n = 4/group. **(B)** Relative mRNA expression of *Calcrl* and *Ramp1* in naïve and activated CD4^+^ T cells treated with PBS or CGRP for 72 h, n = 3–6/group. **(C)** Proliferation of Celltrace blue-labeled activated CD4^+^ T cells following treatment with PBS or CGRP for 24 h, 48 h and 72 h; bar graph showed the percentages of proliferated cells at 72 h, n = 3/group. **(D)** Proliferation of Celltrace blue-labeled activated CD4^+^ T cells isolated from WT mice or *Cd4^Cre^Ramp1-mCherry^fl/fl^* (CD4^Δ^*^Ramp1^*) mice treated with PBS or CGRP for 72 h, n = 3–5/group. **(E)** Mean fluorescence intensities (MFIs) of CD25 on activated CD4^+^ T cells treated with PBS or CGRP for 72 h, n = 3/group. **(F, G)** Concentrations of Th1-type (IL-2, TNF and IFN-γ), Th2-type (IL-4, IL-5, IL-13 and IL-10) and Th17-type (IL-17A) cytokines in culture supernatants, n = 3–4/group. **(H–J)** CD4^+^ T cells were polarized under Th1- **(H)**, Th2-**(I)**, or Th17**(J)**-skewing conditions and treated with PBS or CGRP. MFI of T-bet and percentages of GATA3 and RORγt, percentages of proliferation, and MFI of activation markers (CD69, CD25) were shown, n = 4/group. Graphs depict individual values and group means ± SD **(A–D, F–J)** or means **(E)**. Statistical significance was determined using one-way ANOVA with Tukey’s multiple comparison test **(A, D)** and two–tailed Student’s *t* test **(C–J)** pair: ns, not significant; *P < 0.05; **P < 0.01; ***P < 0.001; ****P < 0.0001.

To investigate the function of CGRP–RAMP1 in CD4^+^ T cells, we first assessed RAMP1 expression during activation. Upon αCD3/αCD28 stimulation, CD4^+^ T cells exhibited marked proliferation with a progressive decrease in RAMP1-mCherry expression (**Figure 2A**). Consistently, *Ramp1* and *Calcrl* transcript levels were downregulated upon activation, and CGRP treatment did not further alter their expression (**Figure 2B**). Moreover, CD4^+^ T cells isolated from medLNs showed *Calcrl* transcript levels comparable to those in splenic CD4^+^ T cells (**Supplementary Figure S5**), supporting the functional relevance of CGRP signaling in this compartment. In contrast, CGRP treatment significantly inhibited CD4^+^ T cell proliferation at 72 hours post-activation (**Figure 2C**). This effect was abolished in *Ramp1*-deficient CD4^+^ T cells, confirming the requirement of RAMP1 for CGRP-mediated suppression, while proliferation remained comparable between *Ramp1^−/−^* and WT CD4^+^ T cells (**Figure 2D**). CGRP exposure also reduced CD25 expression, indicating impaired activation (**Figure 2E**). Consistently, cytokine analysis revealed decreased production of Th1-type (IL-2, TNF and IFN-γ), Th2-type (IL-4, IL-5, IL-13 and IL-10), and Th17-type (IL-17A) cytokines (**Figures 2F, G**). Under lineage-polarizing conditions, CGRP downregulated the expression of T-bet, GATA3, and RORγt, along with reducing proliferation and activation (**Figures 2H–J**). These findings indicate that CGRP constrains CD4^+^ T cell activation and differentiation.

### 3.4 CGRP reshapes the epigenome and transcriptome of CD4^+^ T cells and restrains their Tfh-like differentiation

**Figure 3:**
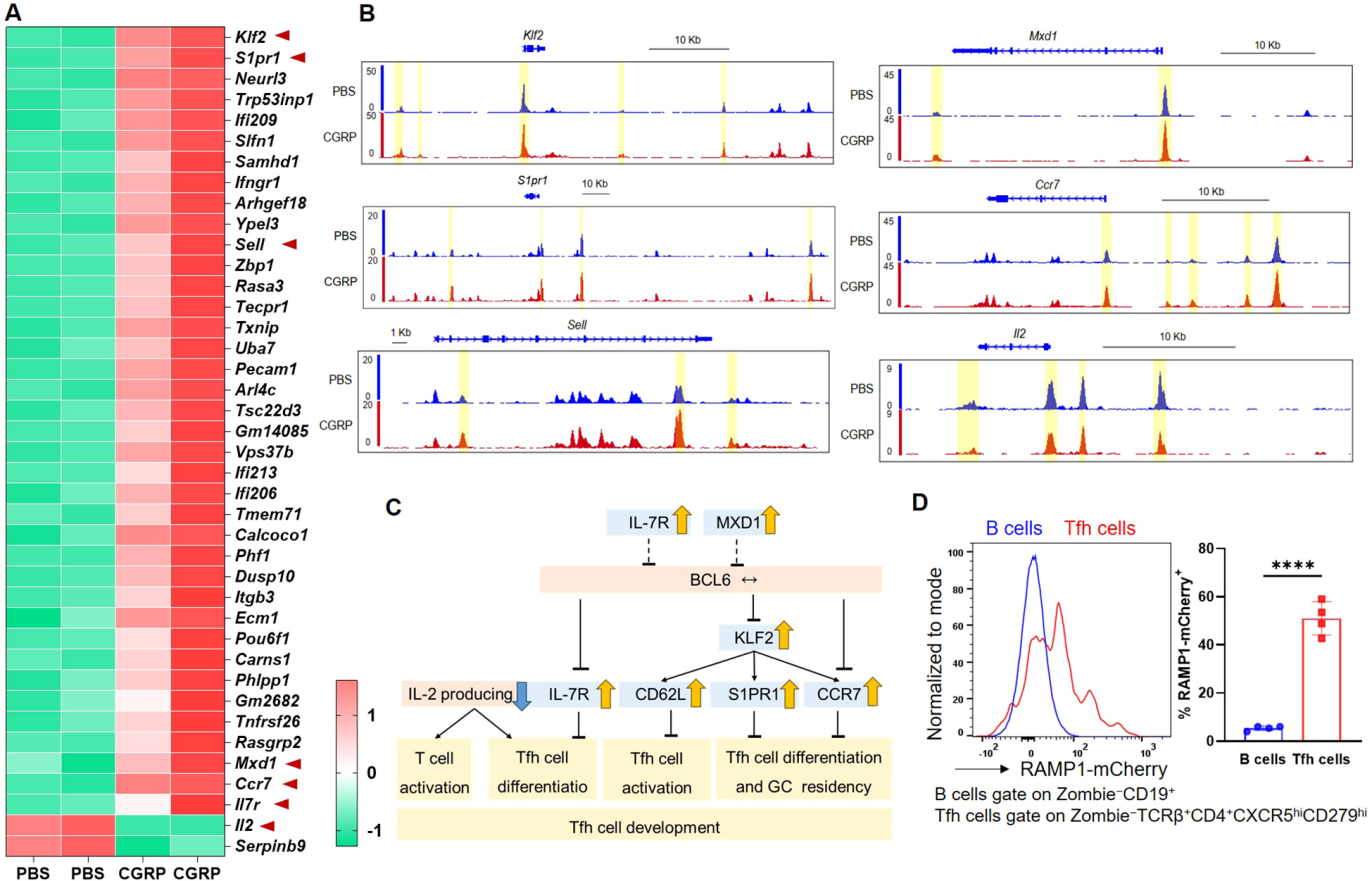
CGRP reshapes chromatin accessibility and transcriptional programs to enforce a hyporesponsive state and repress Tfh-associated gene expression. **(A)** Heatmap of differential expressed genes identified by RNA-seq of CD4^+^ T cells treated with PBS or CGRP. Genes associated with Tfh differentiation or activation (*Klf2*, *S1pr1*, *Sell*, *Mxd1, Ccr7*, *Il7r*, *Il2*) are highlighted, n = 2/group. **(B)** ATAC-seq tracks showing chromatin accessibility of Tfh-related genes (*Klf2*, *S1pr1*, *Sell*, *Mxd1, Ccr7*, *Il2*) in PBS- and CGRP-treated CD4^+^ T cells, n = 2/group. **(C)** Schematic summarizing CGRP-regulated transcriptional programs. Genes induced by CGRP (yellow arrows) or repressed (blue arrows) collectively restrain Tfh activation and differentiation. **(D)** Flow cytometric analysis of the RAMP1 expression in medLN B and Tfh cells from sensitized *Ramp1-mCherry^fl/fl^* mice, n = 4/group. Graph depicts individual values and group means ± SD. Statistical significance was determined using two–tailed Student’s t test: ****P < 0.0001.

To investigate how CGRP modulates CD4^+^ T cell function, we performed transcriptomic and epigenomic profiling of CGRP- versus PBS-treated CD4^+^ T cells. RNA-seq analysis revealed that CGRP treatment altered genes associated with restrained CD4^+^ T cell activation and Tfh differentiation, including downregulation of *Il2 (*28, 29) and upregulation of *Klf2* (30), *S1pr1* (31), *Sell* (32), *Mxd1* (33), *Il7r* (34–36), and *Ccr7* (36) (**Figure 3A**). To assess whether these transcriptional changes were accompanied by epigenetic remodeling, we performed ATAC-seq and observed reduced chromatin accessibility at the *Il2* locus and increased accessibility at *Klf2*, *S1pr1*, *Sell*, *Mxd1*, and *Ccr7* in CGRP-treated cells (**Figure 3B**). By contrast, *Bcl6* transcript levels and accessibility showed no significant changes (**Supplementary Figure S6**), indicating that CGRP does not directly repress *Bcl6* at the RNA or chromatin level in our datasets. These results were integrated into a schematic model (**Figure 3C**), which highlights that CGRP constrains Tfh differentiation by sustaining a naïve-like transcriptional profile and upregulating genes previously linked to the restraint of BCL6-mediated Tfh commitment (28–36) and attenuated IL-2. Consistent with this model, we confirmed that Tfh cells expressed high levels of RAMP1 (**Figure 3D**), establishing them as a relevant target of CGRP–RAMP1 signaling.

Although IL-2 signaling is generally considered antagonistic to Tfh differentiation, it is essential for the initial activation and priming of CD4^+^ T cells toward lineage commitment, including the Tfh fate (29). Supplementation with exogenous IL-2 (25–100 U/mL) restored the proliferative capacity of CGRP-treated CD4^+^ T cells (**Supplementary Figure S7A**), and recovered the expression of the activation markers CD25, CD69, and CD44 (**Supplementary Figure S7B**), suggesting the availability of IL-2 may partially counteract the CGRP-mediated suppression on CD4^+^ T cell activation.

### 3.5 CD4^+^ T cell-specific RAMP1 deficiency promotes Tfh cell accumulation and exacerbates allergic sensitization

To assess whether CGRP directly modulates DC function, we generated BMDCs from WT and *Ramp1-mCherry^fl/fl^* mice using GM-CSF and IL-4. Flow cytometry confirmed RAMP1 expression on BMDCs (**Supplementary Figure S8A**). CGRP treatment did not influence BMDC proliferation (**Supplementary Figure S8B**). Antigen uptake, assessed by DQ-OVA fluorescence at 1 μg/mL and 10 μg/mL, was comparable between CGRP- and PBS-treated BMDCs (**Supplementary Figure S8C**). Similarly, CGRP stimulation had no effect on the expression of MHC-II, CD80, or CD86 (**Supplementary Figure S8D**). To further assess the impact of CGRP on stimulated BMDCs, BMDCs were treated with Poly(I:C), IFN-γ, or GM-CSF in the presence or absence of CGRP. No significant changes were observed in the expression of MHC-II, CD80, or CD86 (**Supplementary Figures S8E, F**). These results indicate that CGRP does not affect the proliferation, antigen uptake, or co-stimulatory phenotype of BMDCs induced by GM-CSF/IL-4, suggesting that its immunoregulatory effects during allergic sensitization are likely mediated through downstream targets such as CD4^+^ T cells, rather than through direct modulation of antigen presentation by DCs.

**Figure 4:**
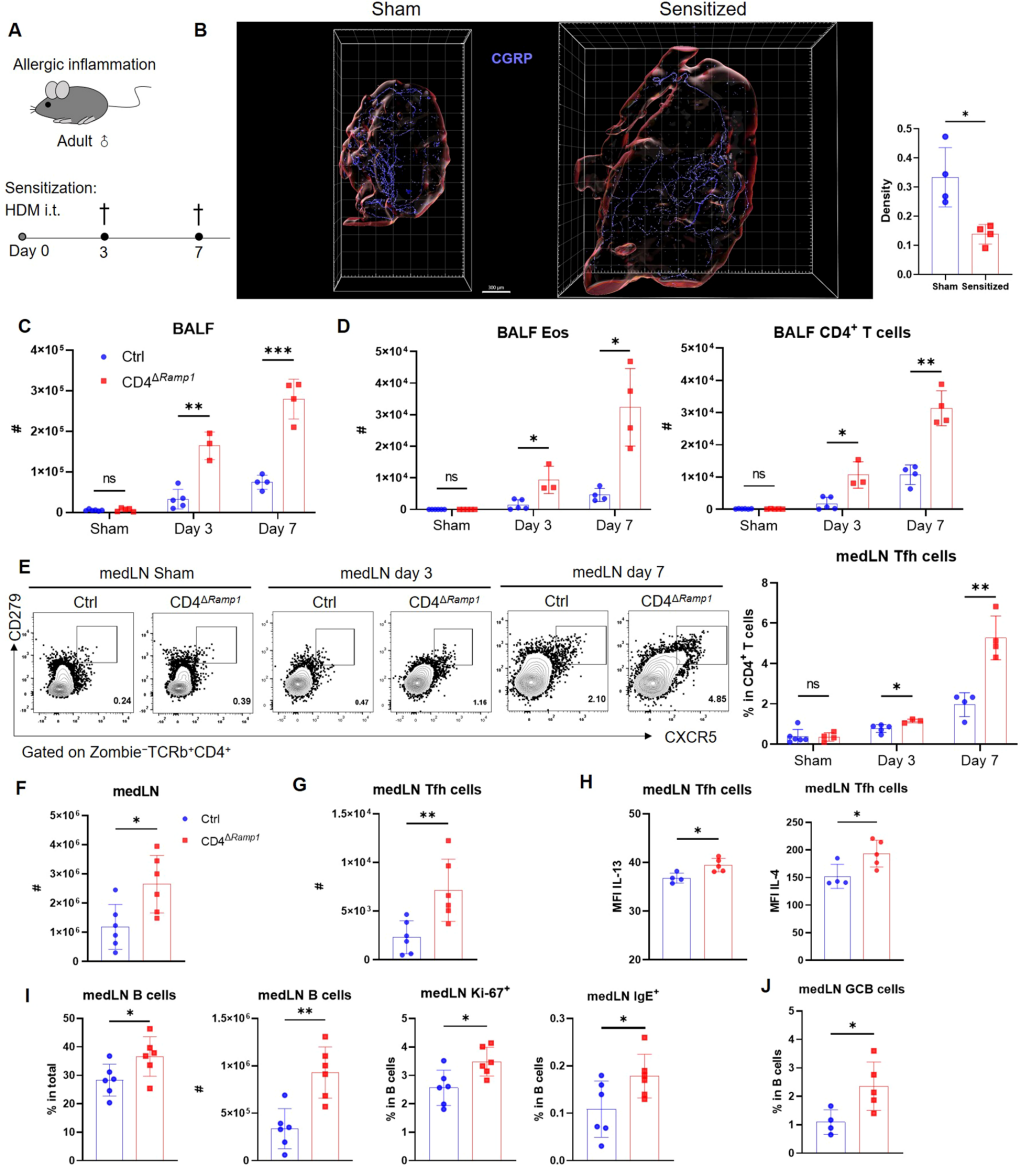
CD4^+^ T cell-specific RAMP1 deficiency promotes Tfh cell accumulation and exacerbates allergic sensitization. **(A)** Adult CD4^Δ^*^Ramp1^* mice and littermate controls (*Cd4^+^Ramp1-mCherry^fl/fl^*, Ctrl) were intratracheally administered HDM to induce lung allergic sensitization. BALF and medLNs were collected for analysis. **(B)** Whole-mount immunohistochemistry and quantification of CGRP^+^ nerve fiber density (CGRP^+^ μm^3^/total tissue volume) showing the distribution of CGRP^+^ fibers (blue) in medLNs from sham and day 7-sensitized mice, n = 4/group. **(C)** Counts of BALF leukocytes on day 3 and 7, n = 3–5/group. **(D)** Counts of BALF eosinophils and CD4^+^ T cells on day 3 and 7, n = 3–5/group. **(E)** Representative contour plots and percentages of medLN Tfh cells on day 3 and 7, n = 3–5/group. **(F)** Counts of medLN leukocytes on day 7, n = 6/group. **(G)** Counts of medLN Tfh cells on day 7, n = 6/group. **(H)** Intracellular cytokine staining of IL-13 and IL-4 in medLN Tfh cells on day 7, n = 4–5/group. **(I)** Percentages and counts of medLN B cells, along with their Ki-67^+^ and IgE^+^ subsets on day 7, n = 6–7/group. **(J)** Frequencies of GC B cells (Zombie^–^CD45^+^B220^+^FAS^+^CD38^–^) in medLN on day 7, n = 4–5/group. Graphs depict individual values and group means ± SD **(B–G)**. Statistical significance was determined using Student’s *t* test with Holm-Sidak correction for multiple comparison **(C–E)**, two–tailed Student’s *t* test **(B, F–J)**: ns, not significant; *P < 0.05; **P < 0.01; ***P < 0.001.

Allergic sensitization begins with Tfh cell activation in draining lymph nodes, preceding Th2-driven airway inflammation (37). We hypothesized that CGRP–RAMP1 signaling in CD4^+^ T cells restrains Tfh responses, thereby limiting subsequent allergic inflammation. To test this hypothesis, we employed an HDM-induced lung sensitization model (**Figure 4A**). Following allergen exposure, CGRP^+^ nerve fiber density in medLNs was reduced (**Figure 4B**; **Supplementary Videos 1**, **2**), and medLNs from sham mice showed an uneven distribution of fibers, with relative enrichment at 40–80 μm beneath the surface and some fibers extending deeper into the interior. Sensitization abolished this enrichment, resulting in a more uniform distribution across depths (**Supplementary Figure S9**). These findings suggest that peripheral sensitization reshapes the spatial organization of CGRP^+^ sensory innervation within medLNs. BALF analysis on days 3 and 7 revealed progressive immune cell infiltration, including eosinophils and CD4^+^ T cells. Compared with littermate controls, CD4^Δ^*^Ramp1^* mice presented an exacerbated airway immune response, suggesting that CGRP–RAMP1 signaling limits CD4^+^ T cell activation during sensitization (**Figures 4C, D**). Notably, CD4^+^ T cell–specific RAMP1 deletion did not significantly affect CD8^+^ T cell frequencies in either BALF or medLNs following HDM sensitization (**Supplementary Figure S10**). Given that Tfh cells initiate allergic sensitization by promoting germinal center formation and IgE class switching in B cells (38), we next examined Tfh responses in medLNs. CD4^Δ^*^Ramp1^* mice presented a time-dependent increase in Tfh cell (CXCR5^+^PD-1^+^) frequencies, with a marked increase on day 7 (**Figure 4E**). Consistent with this, the total numbers of medLN leukocytes, including Tfh cells and B cells, were also elevated (**Figures 4F, G**). Importantly, Tfh-associated cytokines IL-13 and IL-4, which are known to promote IgE class switching and support IgE^+^ B cell survival (37–39), were significantly increased in CD4^Δ^*^Ramp1^* mice compared with controls (**Figure 4H**). In line with this, CD4^Δ^*^Ramp1^* mice also displayed increased B cell proliferation and IgE expression (**Figure 4I**). Furthermore, germinal center (GC) B cells were also significantly expanded in medLNs from CD4^Δ^*^Ramp1^* mice (**Figure 4J**). Together, these results support a model in which CGRP–RAMP1 signaling in CD4^+^ T cells suppresses Tfh accumulation and limits B cell responses, thereby attenuating allergic sensitization.

### 3.6 CGRP suppresses Tfh cell accumulation and limits allergic sensitization

**Figure 5:**
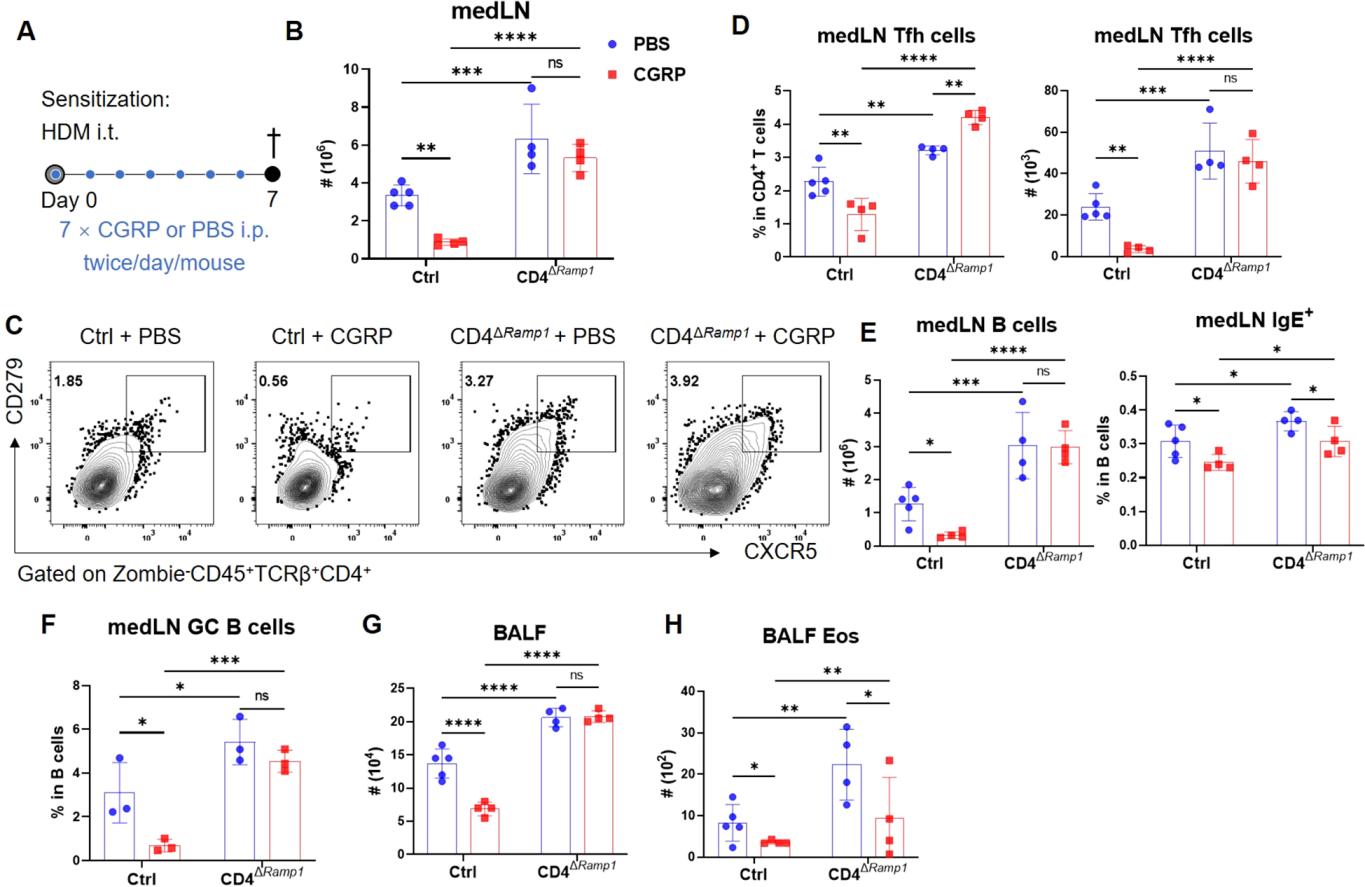
CGRP suppresses Tfh cell accumulation and alleviates allergic sensitization in a RAMP1-dependent manner. **(A)** Adult CD4^Δ^*^Ramp1^* mice and their controls were sensitized with HDM and treated intraperitoneally with CGRP or PBS twice daily from days 0–6. BALF and medLNs were collected on day 7 for flow cytometry analysis. **(B)** Counts of medLN leukocytes, n = 4–5/group. **(C, D)** Representative contour plots **(C)**, percentages and counts of **(D)** medLN Tfh cells, n = 4–5/group. **(E)** Counts of medLN B cells and percentages of IgE^+^ B cells, n = 4–5/group. **(F)** Frequencies of GC B cells in medLNs, n = 3/group. **(G, H)** Counts of BALF leukocytes **(G)** and eosinophils **(H)**, n = 4–5/group. Graphs depict individual values and group means ± SD **(B, D–H)**. Statistical significance was determined using one-way ANOVA with Tukey’s multiple comparison test **(B, D–H)**: ns, not significant; *P < 0.05; **P < 0.01; ***P < 0.001; ****P < 0.0001.

To investigate whether CGRP modulates allergic sensitization by regulating Tfh cells, we administered CGRP intraperitoneally (i.p.) twice daily during the sensitization phase (**Figure 5A**). In control mice, CGRP treatment reduced total medLN leukocyte numbers, as well as both the frequency and absolute number of Tfh cells (**Figures 5B–D**). These effects were not detected in CD4^Δ^*^Ramp1^* mice, indicating that RAMP1 expression on T cells is required for CGRP-mediated suppression of Tfh responses. CGRP also suppressed B cell expansion and reduced the proportion of IgE^+^ B cells in the medLNs of control but not CD4^Δ^*^Ramp1^* mice (**Figure 5E**). Consistently, the frequencies of GC B cells were reduced by CGRP treatment in control but not CD4^Δ^*^Ramp1^* mice (**Figure 5F**). In the airway, CGRP decreased immune cell infiltration and eosinophil accumulation in the BALF, an effect that was diminished in CD4^Δ^*^Ramp1^* mice (**Figures 5G, H**). These findings suggest that CGRP suppresses allergic sensitization by limiting Tfh cell accumulation and downstream B cell responses through a RAMP1-dependent mechanism in CD4^+^ T cells.

### 3.7 RAMP1 expression in CD4^+^ T cells is required to limit allergic asthma

**Figure 6:**
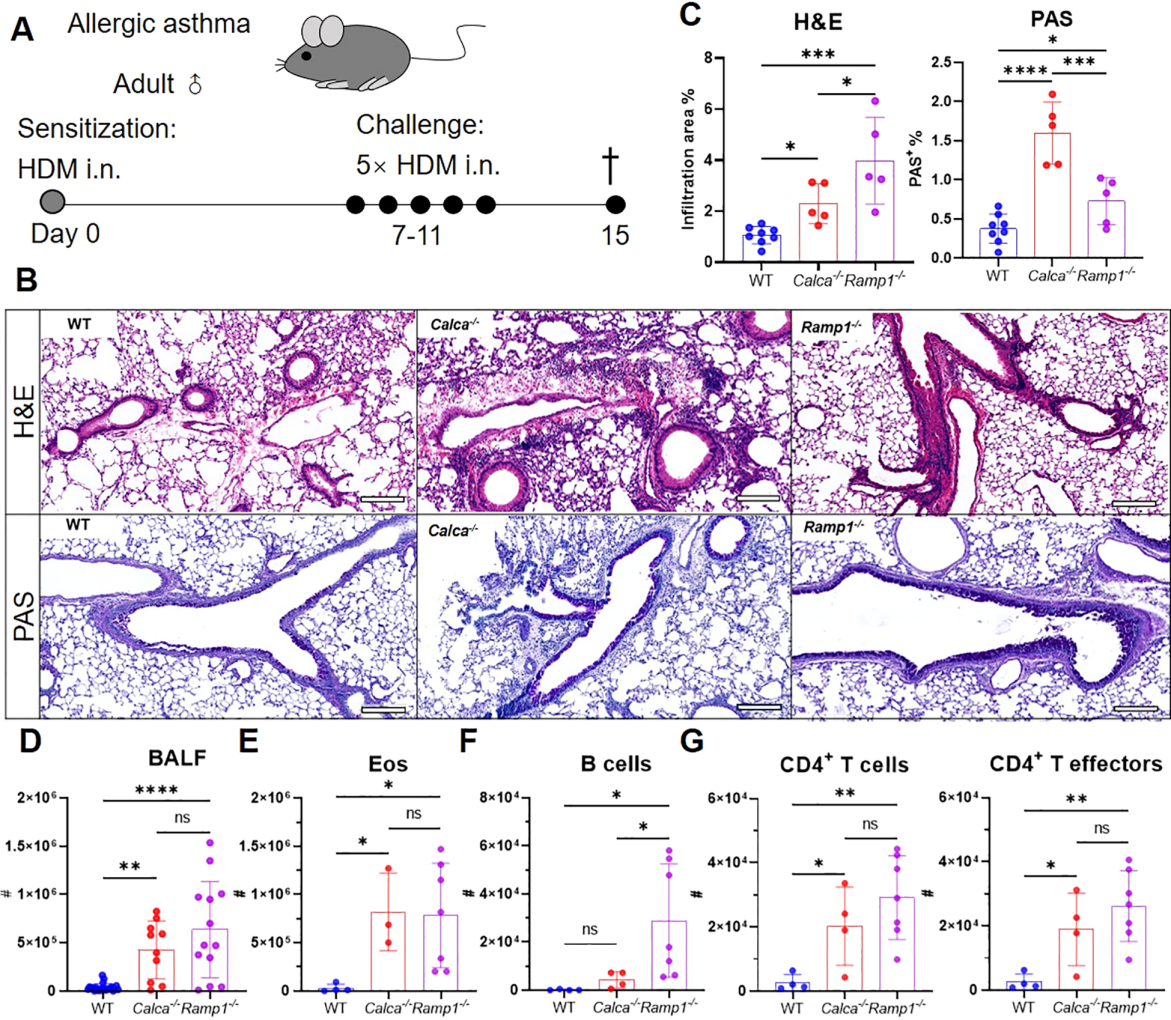
Global loss of *Calca* or *Ramp1* exacerbates allergic asthma. **(A–G)** Adult WT, *Calca^−/−^* and *Ramp1^−/−^* mice and their controls were sensitized intranasally with HDM on day 0 and challenged daily on days 7–11. On day 15, lungs were collected for histopathological analysis, and BALF were harvested for flow cytometry analysis **(A)**. Representative lung sections stained with H&E or PAS **(B)**, and quantification of inflammatory score and PAS^+^ cells **(C)**, scale bars 200 μm, n = 5–8/group. Counts of BALF leukocytes (n = 10–23/group, **D**), eosinophils (n = 3–7/group, **E**), B cells (n = 4–7/group, **F**), CD4^+^ T cells and CD4^+^ T effectors (n = 4–7/group, **G**). Graphs depict individual values and group means ± SD **(C–G)**. Statistical significance was determined using one-way ANOVA with Tukey’s multiple comparison test **(C–G)**: ns, not significant; *P < 0.05; **P < 0.01; ***P < 0.001; ****P < 0.0001.

To investigate the protective role of CGRP–RAMP1 signaling in allergic asthma, we first examined WT, *Ramp1^−/−^* and *Calca^−/−^* mice in an HDM-induced asthma model (**Figure 6A**). On day 15, both strains exhibited exacerbated airway inflammation and mucus hypersecretion, as evidenced by elevated total leukocyte and eosinophil counts, along with increased B cell infiltration in the BALF (**Figures 6B–F**). CD4^+^ T cell numbers were also elevated, predominantly comprising effector subsets (**Figure 6G**), suggesting that CGRP–RAMP1 signaling limits CD4^+^ T cell activation and airway inflammation.

**Figure 7:**
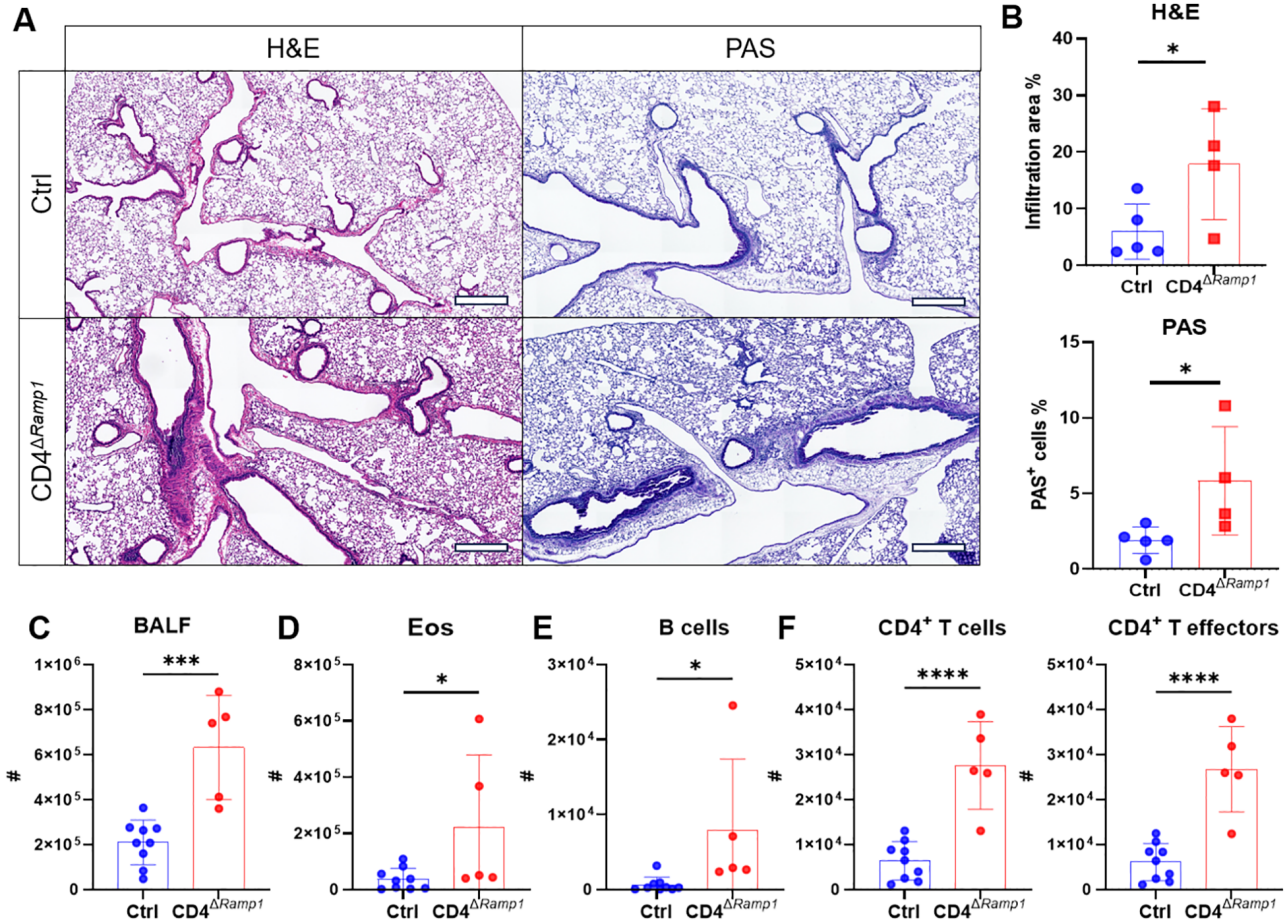
CD4^+^ T cell-specific RAMP1 deficiency aggravates allergic asthma. **(A–F)** Adult CD4^Δ^*^Ramp1^*mice and their controls were intranasally injected with HDM to induce allergic asthma. On day 15, lungs were collected for histopathological analysis, and BALF were harvested for flow cytometry analysis. Representative lung sections stained with H&E or PAS **(A)**, and quantification of inflammatory score and PAS^+^ cells **(B)**, scale bars 400 μm, n = 4–5/group. Counts of BALF leukocytes **(C)**, eosinophils **(D)**, B cells **(E)**, CD4^+^ T cells and CD4^+^ T effectors **(F)**, n = 5–9/group. Graphs depict individual values and group means ± SD **(B–F)**. Statistical significance was determined using two–tailed Student’s *t* test **(B–F)**: *P < 0.05; ***P < 0.001; ****P < 0.0001.

Given these findings, we next assessed the CD4^+^ T cell–specific contribution of RAMP1 by subjecting CD4^Δ^*^Ramp1^* mice and their littermate controls to HDM sensitization and challenge. Compared with littermate controls, CD4^Δ^*^Ramp1^* mice displayed exacerbated airway inflammation and mucus hypersecretion, with increased leukocyte eosinophil and B cell counts in the BALF (**Figures 7A–E**). CD4^+^ T cells were also expanded, with a marked increase in effector subsets (**Figure 7F**). These findings suggest that RAMP1 expression in CD4^+^ T cells is required for CGRP-mediated suppression of allergic inflammation.

### 3.8 CGRP treatment during sensitization attenuates allergic asthma

**Figure 8:**
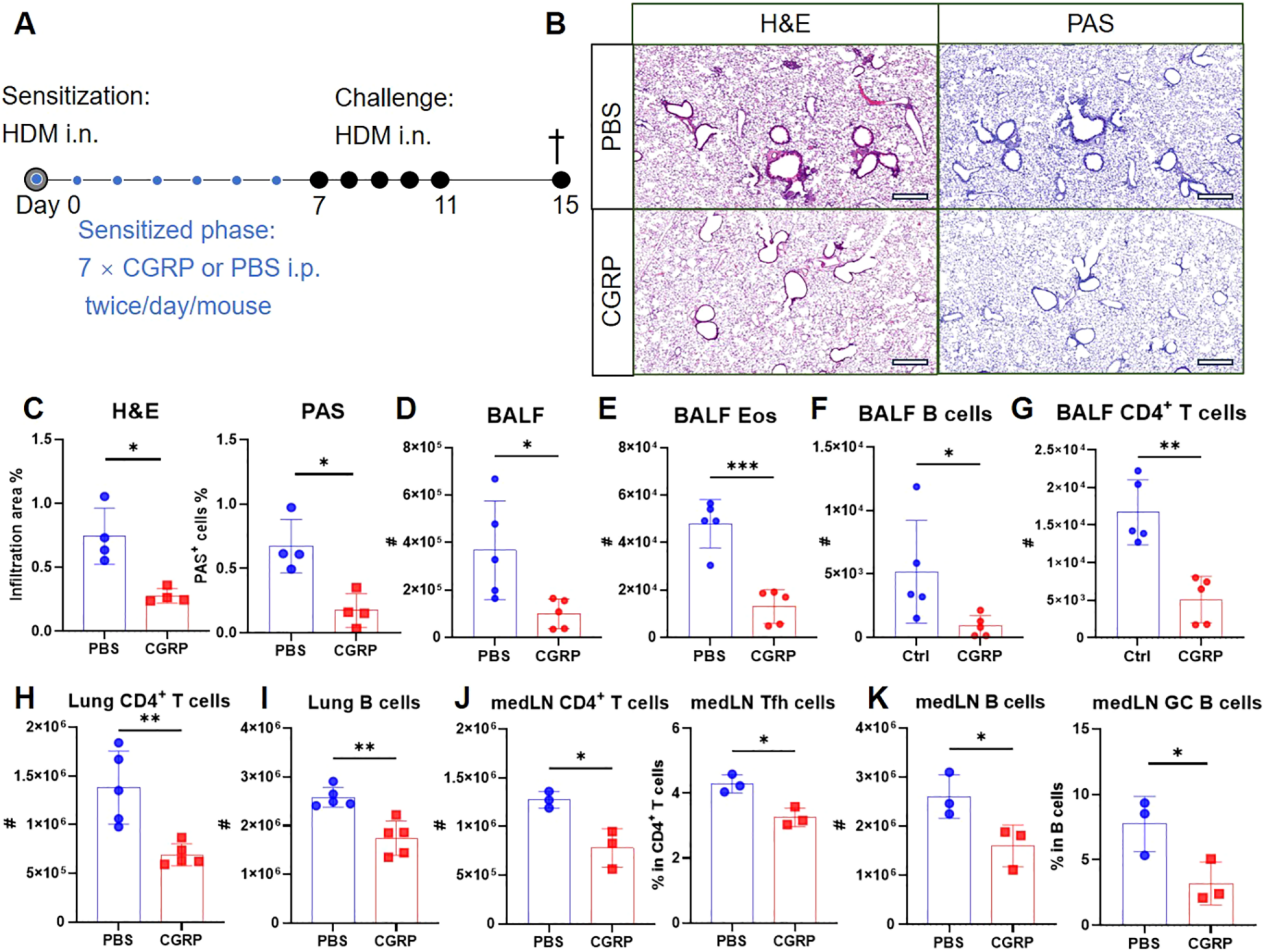
CGRP treatment during sensitization attenuates allergic asthma. **(A–K)** Adult mice were intranasally administered with HDM to induce allergic asthma, and treated intraperitoneally with CGRP or PBS twice daily from days 0–6. On day 15, lungs were collected for histopathological analysis, and BALF, lungs and medLNs were harvested for flow cytometry analysis **(A)**. Representative lung sections stained with H&E or PAS **(B)**, and quantification of inflammatory score and PAS^+^ cells **(C)**, scale bars 400 μm, n = 4/group. Counts of BALF leukocytes **(D)**, eosinophils **(E)**, B cells **(F)** and CD4^+^ T cells **(G)**, n = 4–5/group. Counts of CD4^+^ T cells **(H)** and B cells **(I)** in the lungs, n = 4–5/group. Counts of CD4^+^ T cells and frequencies of Tfh cells in medLNs, n = 3/group **(J)**. Counts of B cells and frequencies of GC B cells in medLNs, n = 3/group **(K)**. Graphs depict individual values and group means ± SD **(C–K)**. Statistical significance was determined using two–tailed Student’s *t* test **(C–K)**: ns, not significant; *P < 0.05; **P < 0.01; ***P < 0.001.

Given that RAMP1 expression in CD4^+^ T cells is required for CGRP-mediated protection, we next examined whether exogenous CGRP administration during sensitization could recapitulate this protective effect. Mice were administered CGRP intraperitoneally twice daily throughout the sensitization phase (**Figure 8A**). CGRP treatment reduced lung inflammation and mucus production (**Figures 8B, C**), with decreased numbers of leukocytes, eosinophils and B cells in the BALF (**Figures 8D–F**). CD4^+^ T cell accumulation was also suppressed (**Figure 8G**). Flow cytometric analysis further revealed that CGRP treatment reduced the numbers of CD4^+^ T cells and B cells in both lungs and medLNs (**Figures 8H–K**). The relative frequencies of these subsets remained unchanged, except for lung CD4^+^ T cells, which were reduced (**Supplementary Figures S11A, B**). In addition, CGRP suppressed the frequencies of Tfh cells and GC B cells in medLNs (**Figures 8J, K**). By contrast, CXCR5^hi^CD279^hi^ Tfh cells were barely detectable in lung tissue (**Supplementary Figure S9**), consistent with previous reports that Tfh cells are restricted to draining LNs and give rise to Th2 effector populations that migrate to the lung (37). Together, these findings suggest a model in which CGRP alleviates allergic asthma primarily through RAMP1-mediated regulation of CD4^+^ T cells during the sensitization phase.

## 4 Discussion

Neuroimmune communication has emerged as a critical layer of immune regulation, yet how sensory neuropeptides precisely shape adaptive immune responses to maintain tissue homeostasis and restrain pathological inflammation remains unclear. Neuron-derived cues may constitute an underappreciated axis that complements conventional anti-inflammatory strategies.

CGRP, a sensory neuropeptide, is increasingly recognized as an important regulator of immune responses. While RAMP1 is abundantly expressed throughout thymocyte maturation, both our data (not shown) and those of prior studies (40) confirmed the presence of CGRP in the thymus, *Ramp1* or *Calca* deletion did not alter the thymocyte composition or peripheral T cell seeding. Moreover, it did not affect TCR rearrangement, suggesting that RAMP1 is dispensable for thymic T cell development under homeostatic conditions. However, previous studies have shown that CGRP can prevent aberrant thymocyte activation and that its expression decreases with age (41), suggesting that RAMP1 function may become more relevant under conditions of inflammation or aging. This could reflect either the intrinsically low signaling activity of the CGRP–RAMP1 axis in the steady-state thymus (42) or functional redundancy with other developmental pathways. Notably, RAMP1 expression on both thymocytes and peripheral CD4^+^ T cells appear to be specifically tuned to respond to CGRP. In contrast, the expression of CALCR, the coreceptor for IAPP (43), was virtually undetectable in these populations (data not shown), supporting a selective role for CGRP–RAMP1 signaling in T cell biology.

Evidence from both human and murine studies implicates sensory neurons in allergic airway diseases. CGRP^+^ PNECs are markedly increased in the lungs of asthma patients (44), and ablation of sensory neurons mitigates OVA-induced allergic inflammation in mice, highlighting the importance of neuroimmune crosstalk (45, 46). In our model, HDM sensitization reduced the density of CGRP^+^ fibers in medLNs, while CGRP inhibited CD4^+^ T cell responses and allergic sensitization. Consistent with earlier reports showing that sensory nerves rarely penetrate the deep cortical region of popliteal and inguinal lymph nodes (5), CGRP^+^ fibers in medLNs were mostly enriched in superficial regions. Nevertheless, a subset of fibers extended deeper into the parenchyma, thereby facilitating potential interactions with CD4^+^ T cells. Notably, Nav1.8^+^ sensory fibers have been shown to closely associate with subcapsular macrophages and naïve lymphocytes (7), suggesting that neuronal inputs may influence both innate and adaptive immune compartments. Furthermore, although Tfh cells classically reside in secondary lymphoid organs where they support GC B cell responses, Tfh-like populations have been detected in lymph and blood (47), raising the possibility that these cells, during their transit through lymph nodes, could be exposed to sensory neuron-derived CGRP in subcapsular or medullary regions. Such spatial proximity may provide a route by which CGRP signaling modulates Tfh differentiation, maintenance, or egress into circulation. Interestingly, HDM stimulation activates TRPV1^+^ nociceptors, triggering SP release while suppressing CGRP (48). This imbalance may tilt the local immune microenvironment toward allergic sensitization. Our study revealed that CGRP suppresses allergic sensitization and the development of allergic asthma, which is consistent with previous findings (49). In contrast, SP has been shown to induce the migration of CD301b^+^ dendritic cells (48) or trigger mast cell degranulation (50), thereby initiating allergic responses. These findings underscore the neuropeptide specific-, context-dependent roles of sensory neurons in orchestrating allergic inflammation.

While RAMP1 expression is detected across multiple immune cell types, including CD4^+^ and CD8^+^ T cells, alveolar macrophages, neutrophils, eosinophils, DCs, and ILCs, our focus is CD4^+^ T cells, which express high RAMP1 levels and play a pivotal role in allergic sensitization (51). Despite their key role in initiating type 2 immunity, DCs in our system (GM-CSF/IL-4-induced BMDCs) showed no CGRP-mediated alterations in proliferation, antigen uptake, or the expression of MHC II and costimulatory molecules. This finding contrasts with reports in cDC1s where CGRP enhanced CD8^+^ T cell activation via cross-presentation, likely reflecting subset-specific roles (52). Furthermore, CGRP inhibits antigen presentation by epidermal Langerhans cells, and DCs are intimately associated with sensory nerves, suggesting tissue and context specificity (53). Thus, in allergic sensitization, CGRP likely modulates CD4^+^ T cell activation and differentiation more directly than through classical cDC2 antigen presentation.

Our data indicate that CGRP suppresses CD4^+^ T cell polarization toward the Tfh lineage, a pivotal step in initiating allergic sensitization. While Tfh cells do not directly mediate airway inflammation, they orchestrate IgE production by B cells and serve as precursors for pathogenic Th2 cells upon repeated allergen exposure (37). Mechanistically, we observed that CGRP upregulated *Klf2*, *S1pr1*, *Sell*, and *Ccr7*—genes that sustain the naïve T cell program and inhibit Tfh commitment (30, 32, 54). In contrast, canonical Tfh markers such as CXCR5 and PD-1 are induced during follicular migration and B cell interactions (54). In addition, CGRP increased the expression of *Mxd1* and *Il7r*, both of which antagonize BCL6 signaling (33, 34, 54), thereby reinforcing transcriptional networks that constrain Tfh differentiation. By contrast, *Bcl6* transcript levels and chromatin accessibility were not significantly altered following CGRP treatment. These findings do not support a mechanism in which CGRP directly represses *Bcl6* transcription but instead suggest that it modulates BCL6-associated downstream pathways. Nevertheless, the possibility that CGRP influences BCL6 at the protein or post-transcriptional level cannot be excluded and warrants further investigation. Although Tfh-like cells can be generated *in vitro* under specific cytokine conditions (35, 55, 56), their differentiation efficiency and phenotypic stability remain variable across protocols, and no standardized system has been established (57, 58). Therefore, we did not pursue CGRP-based *in vitro* Tfh skewing assays in this study.

Our study suggests that CGRP is a negative regulator of Tfh cell differentiation. Although IL-2 is known to inhibit Tfh differentiation via STAT5-BLIMP1 signaling (36), emerging evidence suggests a more nuanced model in which IL-2-producing CD4^+^ T cells can themselves commit to the Tfh lineage while limiting neighboring cells through paracrine IL-2 signaling (28). Importantly, IL-2 is also indispensable for the priming and survival of naïve CD4^+^ T cells (29, 59). This dual function highlights a temporal dichotomy: IL-2 is essential for early activation but must be silenced to permit Tfh commitment. Thus, by dampening IL-2 production, CGRP may limit the expansion of CD4^+^ T cells and restrict the pool of cells available for Tfh polarization. Beyond IL-2, Tfh-associated cytokines represent another key regulatory axis in allergic sensitization. Although IL-21 is a hallmark Tfh cytokine, its role in IgE regulation is context-dependent and species-specific (60, 61). In contrast, IL-4 and IL-13 are consistently implicated in sustaining IgE^+^ B cell survival and enhancing IgE production, and Tfh subsets producing these cytokines have been well documented (37–39). In line with this, our analysis revealed increased IL-4 and IL-13 in CD4*^ΔRamp1^* mice, supporting the contribution of Tfh-derived cytokines to B cell responses during allergic sensitization.

Furthermore, CGRP may dampen Th2 cell development, which is consistent with recent findings that CGRP–RAMP3 signaling attenuated IL-13 production during viral infection (62). These results suggest that CGRP broadly modulates CD4^+^ T cell subset differentiation, underscoring the need to dissect receptor-specific signaling cascades in future studies. In addition, while our data identify CD4^+^ T cells as primary targets of CGRP–RAMP1 signaling the suppression of allergic sensitization, the partial loss of protection observed in T cell-specific RAMP1-deficient mice implies contributions from additional cell types. Previous studies have shown that CGRP can also regulate ILC2s (12) and endothelial cells (63), suggesting that these populations may cooperate with CD4^+^ T cells to mediate the inflammatory effects induced by CGRP. CGRP exerts potent vasodilatory effects (64). *In vitro*, 100 nM CGRP is a commonly used concentration for receptor activation assays (12, 48, 65–67). Previous studies indicate that concentrations above 100 nM can elicit robust vascular responses but do not influence protein extravasation or sensory irritation (64, 68). *In vivo*, CGRP doses 1–10 µg/mouse/day have been used to inhibit influenza, airway inflammation, colitis and liver injury (12, 65–67). Considering the short half-life (7–10 min) of CGRP in adult circulation (69), we employed 5 µg/mouse twice a day and did not find any adverse effect. Collectively, both the *in vitro* and *in vivo* doses used in our study are consistent with the ones widely used and experimentally applicable.

Our results highlight CD4^+^ T cells as key mediators of the effects of CGRP–RAMP1 on allergic sensitization. CGRP–RAMP1 may thus represent a complementary approach to existing allergic asthma therapies. In contrast to the broad-spectrum inflammatory effects and associated adverse outcomes of corticosteroids (70, 71), CGRP selectively targets neuroimmune interactions. Integrating CGRP-based interventions with conventional treatments may provide novel therapeutic opportunities, particularly for patients with steroid-resistant or severe asthma.

Although serum IgE is often regarded as a useful marker of allergic responses, many murine allergic asthma models validate primarily through histopathology, BALF analyses, and lung cellular responses without including IgE measurements (44, 72–79). In line with these practices, we assessed H&E and PAS staining, BALF immune cell infiltration, and LN-derived B cell and GC responses—which demonstrated enhanced sensitization and airway inflammation, as well as their attenuation under CGRP–RAMP1 signaling. Future studies incorporating serological IgE analyses will provide an additional layer of validation and further refine these findings. In addition, a limitation of our study is that the CD4-Cre system also induces recombination in CD8^+^ T cells due to transient CD4 expression during thymic development. However, CD4^+^ T cells are well established as the principal drivers of allergic sensitization and asthma, whereas the involvement of CD8^+^ T cells has not been widely recognized. Consistent with this, our data showed that CD4^+^ T cell–specific RAMP1 deletion did not significantly alter CD8^+^ T cell populations, suggesting that the phenotypes described here are predominantly mediated by CD4^+^ T cells.

## 5 Conclusion

Our study identifies the CGRP–RAMP1 pathway as a neuroimmune axis that plays a pivotal role in regulating adaptive immune responses, particularly in allergic sensitization. We demonstrate that CGRP^+^ sensory nerve fibers densely innervate medLNs, and their density significantly decreases after allergen sensitization. By showing that RAMP1 is preferentially expressed on naïve CD4^+^ T cells, we uncover a mechanism by which CGRP, through RAMP1, modulates T cell activation and differentiation. Notably, exogenous CGRP treatment attenuates Tfh cell accumulation and B cell activation, mitigating allergic sensitization in a RAMP1-dependent manner. Furthermore, our *in vivo* asthma model shows that systemic CGRP treatment during the sensitization reduces airway inflammation and mucus hypersecretion. These findings highlight the importance of neuroimmune regulation in allergic diseases and identify the CGRP–RAMP1 axis as a potential therapeutic target linking the nervous and immune systems.

## Data Availability

Data will be made available on request. The raw sequencing data from this study have been deposited in the Genome Sequence Archive in BIG Data Center (https://bigd.big.ac.cn/), Beijing Institute of Genomics (BIG), Chinese Academy of Sciences, under the accession number: TCR-seq, PRJCA046607; ATAC-seq, PRJCA046606; and RNA-seq, PRJCA046604.

## Supplementary material

Supplementary Video 1 Whole-mount immunohistochemistry showing the distribution of CGRP^+^ fibers (blue) in medLNs from a representative sham mouse.

Supplementary Video 2 Whole-mount immunohistochemistry showing the distribution of CGRP^+^ fibers (blue) in medLNs from a representative day 7-sensitized mouse.
