# Supplementary material for: CGRP restrains CD4+ T cell responses and allergic sensitization
Source: Front Immunol. 2025 Nov 18;16:1671269. doi: 10.3389/fimmu.2025.1671269 (PMC12746649; doi:10.3389/fimmu.2025.1671269)
Supplement: Supplementary file 3 [file DataSheet1.pdf]

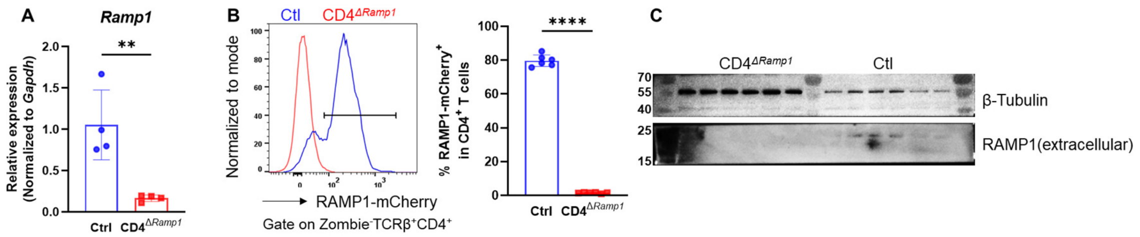

Figure S1. Validation of RAMP1 deletion in CD4$^+$ T cells from CD4$^{\Delta Ramp1}$ mice.
(A) Relative *Ramp1* mRNA expression in splenic CD4$^+$ T cells isolated from CD4$^{\Delta Ramp1}$ mice and their littermate controls (*Cd4$^+$Ramp1-mCherry$^{fl/fl}$*), n = 4/group. **(B)** Flow cytometric analysis of the RAMP1-mCherry expression in medLN CD4$^+$ T cells from CD4$^{\Delta Ramp1}$ mice and controls, n = 6/group. **(C)** Extracellular RAMP1 expression on in splenic CD4$^+$ T cells from CD4$^{\Delta Ramp1}$ mice and controls, assessed by immunoblotting, n = 6/group. Graphs depict individual values and group means ± SD **(A–B)**. Statistical significance was determined using two–tailed Student's *t* test **(A–B)**: ns, not significant; **P < 0.01; ****P < 0.0001.

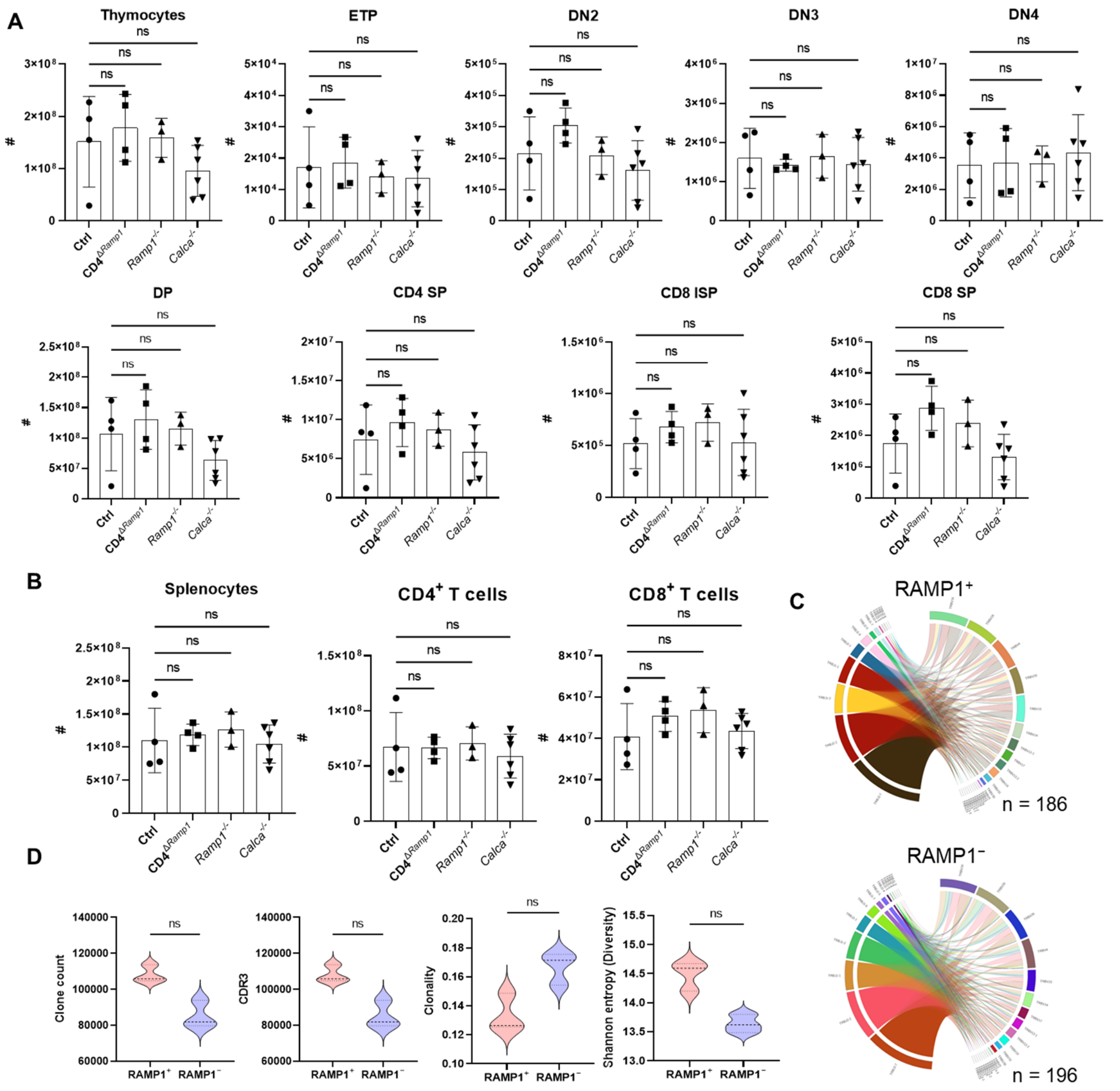

Figure S2. RAMP1 does not affect thymocyte development, peripheral T cell seeding and TCR diversity.

(A) Numbers of thymocytes in thymus from CD4$^{\Delta Ramp1}$, *Ramp1*$^{-/-}$, *Calca*$^{-/-}$ mice and their controls (Ctrl), n = 4–6/group; **(B)** Numbers of splenocytes, CD4^{+} and CD8^{+} T cell in spleens from CD4$^{\Delta Ramp1}$, *Ramp1*$^{-/-}$, *Calca*$^{-/-}$ mice and their Ctrl, n = 4–6/group; **(C–D)** TCR-seq for RAMP1-mCherry^{+} and RAMP1-mCherry^{-} CD4^{+} T cells sorted from spleens of *Ramp1-mCherry*$^{fl/fl}$ mice (n = 3/group, from 6 mice). Splenic circos plots showing TCR V–J gene usage of RAMP1^{+} and RAMP1^{-} CD4^{+} T cells sorted from spleens of *Ramp1-mCherry*$^{fl/fl}$ mice **(C)**. Quantification of TCR diversity based on clone count, CDR3 count, clonality, and Shannon index between RAMP1-mCherry^{+} and RAMP1-mCherry^{-} CD4^{+} T cells **(D)**. Graphs depict individual values and group means ± SD **(A–B)**. Statistical significance was determined using one-way ANOVA with Tukey's multiple comparison test **(A–B)**: ns, not significant.

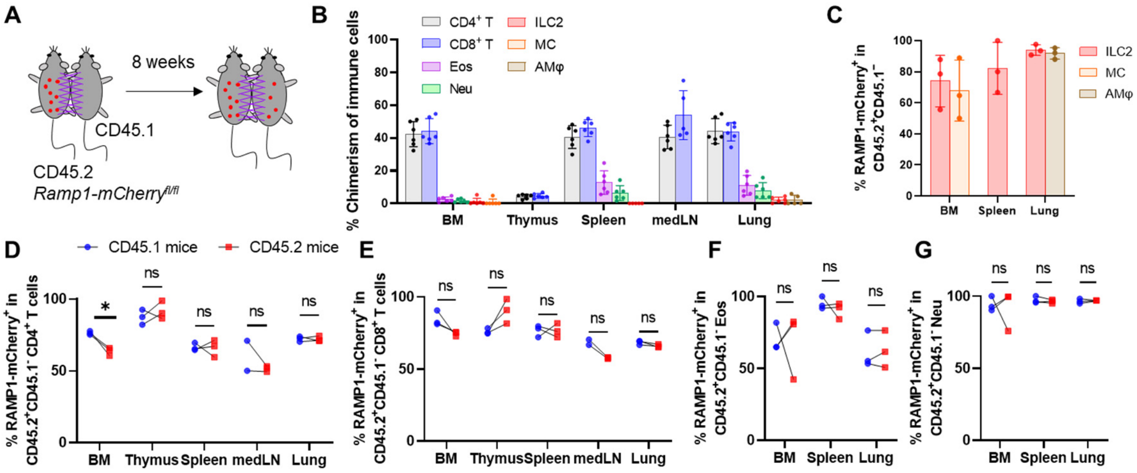

Figure S3. RAMP1 does not affect tissue residency of leukocytes.

(A) Schematic of parabiosis experiment. CD45.2 *Ramp1-mCherry*$^{fl/fl}$ mice were surgically conjoined to CD45.1 WT mice. After 8 wk, BM, thymus, spleen, medLN and lung for flow cytometric analysis, n = 2–3 parabiontic pairs, one representative of two independent experiments. **(B)** Percentages of partner-derived immune cells after separation of the parabionts, including CD4^{+} T cells, CD8^{+} T cells, eosinophils, neutrophils, ST2^{+} ILC2s, mast cells (MC, Zombie^{-}CD11b^{+}CD11c^{-}FceR1^{+}ST2^{+}), and AMφ. **(C)** Percentages of RAMP1-mCherry^{+} in ILC2s, mast cells and AMφ of CD45.2 *Ramp1-mCherry*$^{fl/fl}$ mice. **(D–G)** Percentages of RAMP1-mCherry^{+} in CD4^{+} T cells **(D)**, CD8^{+} T cells **(E)**, eosinophils **(F)** and neutrophils **(G)** of CD45.1 WT and CD45.2 *Ramp1-mCherry*$^{fl/fl}$ mice. Graphs depict individual values and group means ± SD **(B, G)**. Statistical significance was determined using two–tailed paired Student's *t* test **(C–F)**: ns, not significant; *P < 0.05.

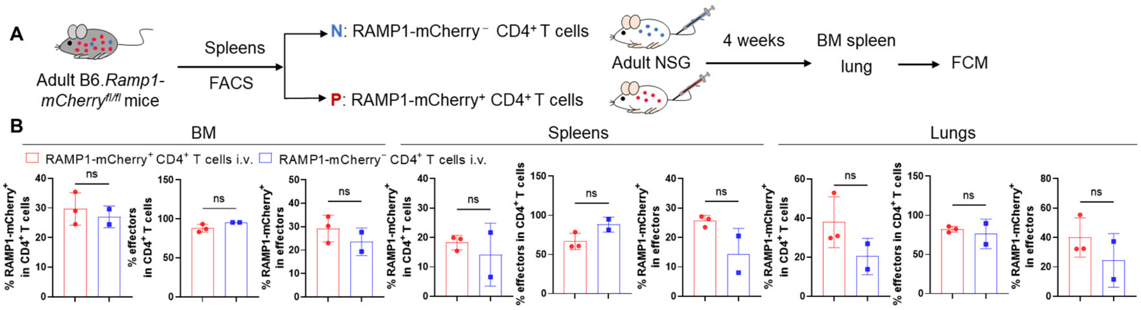

Figure S4. RAMP1 expression is dynamically regulated in CD4$^+$ T cells.

(A) Schematic of adoptive transfer of sorted RAMP1-mCherry$^+$ or RAMP1-mCherry$^-$ CD4$^+$ T cells into adult NSG mice and flow cytometer analysis after 4 wk. **(B)** Percentages of RAMP1-mCherry$^+$ in engrafted CD4$^+$ T cells of BM, spleens and lungs, n = 2–3/group. Graphs depict individual values and group means ± SD. Statistical significance was determined using two–tailed Student's *t* test: ns, not significant.

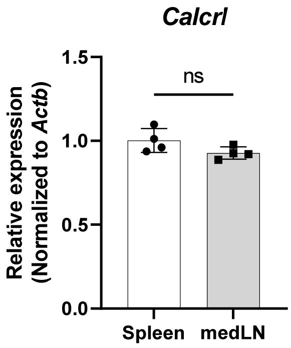

Figure S5. *Calcrl* expression in CD4$^+$ T cells from medLNs and spleens.
Relative mRNA expression of *Calcrl* in CD4$^+$ T cells isolated from spleens and medLNs (n = 4/group). Graphs depict individual values and group means ± SD. Statistical significance was determined using two–tailed Student's *t* test: ns, not significant.

A

Gene Name	Padj	PBS	PBS	CGRP	CGRP
Bcl6	0.08	410	455	630	703

B

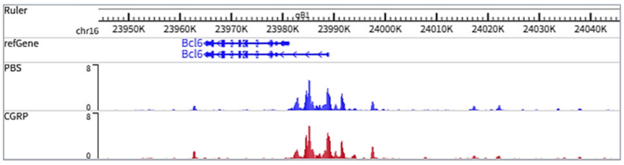

Figure S6. *Bcl6* transcript levels and chromatin accessibility are not significantly altered by CGRP treatment in CD4$^+$ T cells.

(A) RNA-seq analysis of *Bcl6* expression in CD4$^+$ T cells treated with PBS or CGRP, n = 2/group. **(B)** ATAC-seq tracks showing chromatin accessibility of the *Bcl6* locus in PBS- and CGRP-treated CD4$^+$ T cells, n = 2/group.

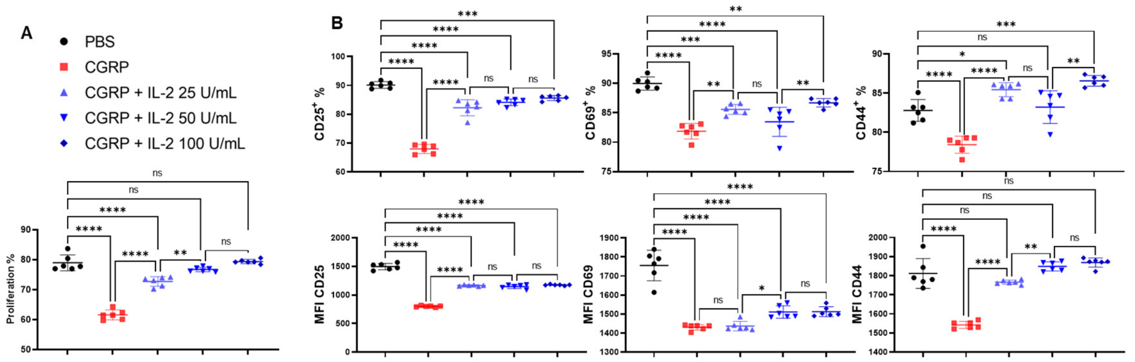

Figure S7. IL-2 restores CD4^{+} T cell activation suppressed by CGRP.

(A–B) Activated CD4^{+} T cells were stimulated with PBS, CGRP, or CGRP combined with IL-2 at 25, 50, or 100 U/mL. Percentages of proliferation **(A)**, and percentages and MFI of activation markers CD25, CD69 and CD44 **(B)** were assessed by flow cytometry. Graphs depict individual values and group means ± SD. Statistical significance was determined using one-way ANOVA with Tukey's multiple comparison test: ns, not significant; *$P < 0.05$; **$P < 0.01$; ***$P < 0.001$; ****$P < 0.0001$.

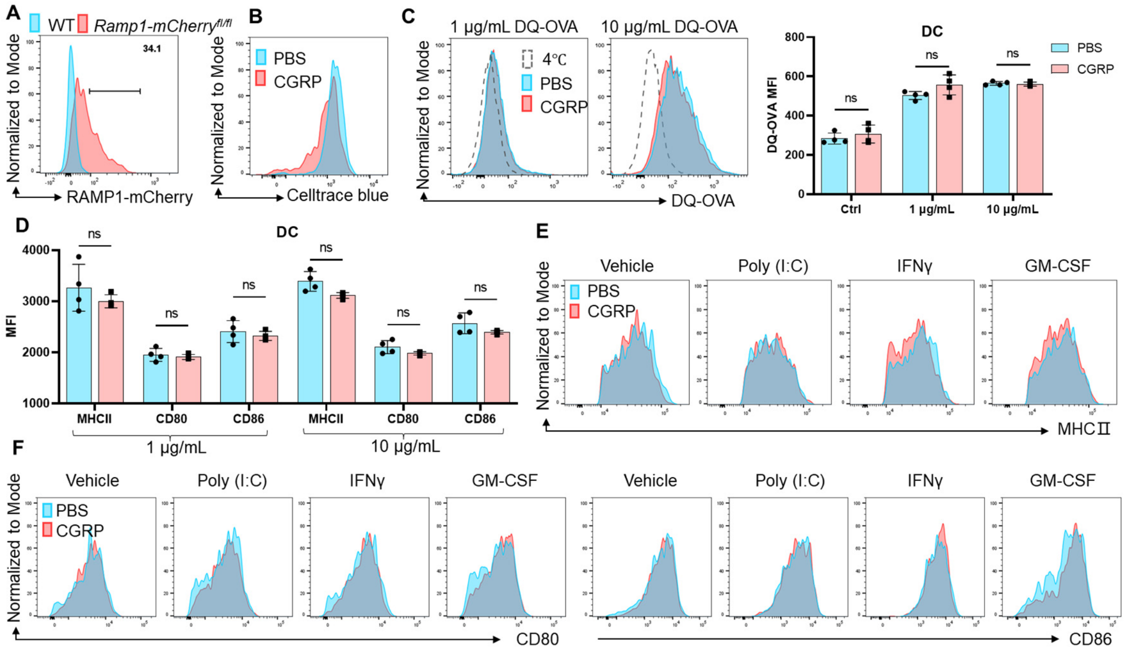

Figure S8. CGRP does not alter antigen uptake or activation phenotype in GM-CSF/IL-4–derived BMDCs.

(A) Representative histogram of RAMP1 expression in BMDCs differentiated from bone marrow cells of WT or *Ramp1-mCherry*$^{fl/fl}$ mice with GM-CSF and IL-4, n = 8/group. **(B)** Proliferation of Celltrace blue-labeled BMDCs cultured with PBS or CGRP, n = 3/group. **(C–D)** Antigen processing was measured by incubating BMDCs with 1 μg/mL or 10 μg/mL DQ-OVA in the presence or absence of CGRP. Samples incubated at 4°C served as negative control, n = 4/group. MFI of processed DQ-OVA **(C)** and MHCII, CD80 and CD86 expression **(D)** on BMDCs following PBS or CGRP treatment**. (E–F)** Overlay histograms showing MHCII **(E)**, CD80 and CD86 **(F)** expression on BMDCs stimulated with vehicle, Poly(I:C), IFN-γ, or GM-CSF, and treated with PBS or CGRP, n = 4/group. Graphs depict individual values and group means ± SD **(C–D)**. Statistical significance was determined using Student's *t* test with Holm-Sidak correction for multiple comparison **(C–D)**: ns, not significant.

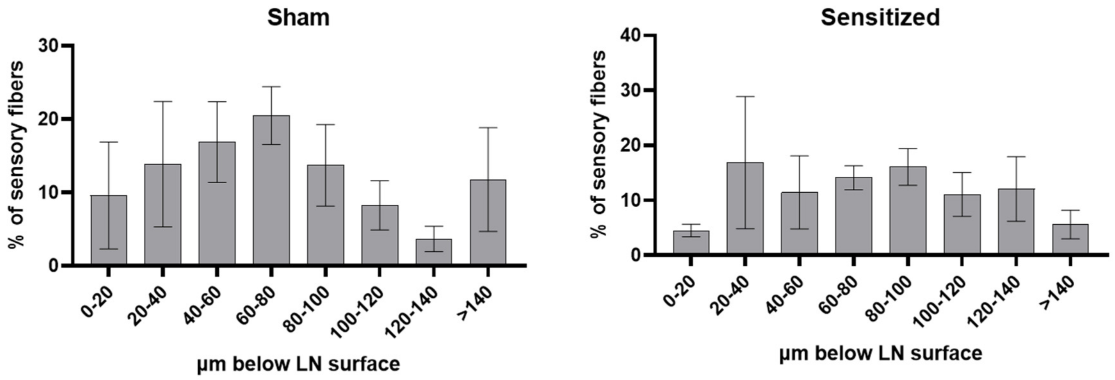

Figure S9. Spatial distribution of CGRP$^+$ nerve fibers in medLNs.

Whole-mount immunohistochemistry and quantification of the penetration depth of CGRP$^+$ nerve fibers as a percentage of total intranodal sensory fibers in medLNs from sham and day 7-sensitized mice, n = 4/group. Graphs depict individual values and group means ± SEM.

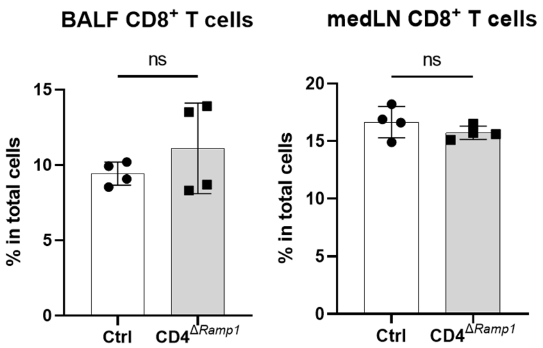

Figure S10. RAMP1 deletion in T cells does not alter CD8$^+$ T cell frequencies in allergic sensitization.

Adult CD4$^{\Delta Ramp1}$ mice and controls were intratracheally administered HDM to induce lung allergic sensitization. BALF and medLNs were collected for CD8$^+$ T cell flow cytometric analysis, n = 4/group. Graphs depict individual values and group means ± SD. Statistical significance was determined using two–tailed Student's *t* test: ns, not significant.

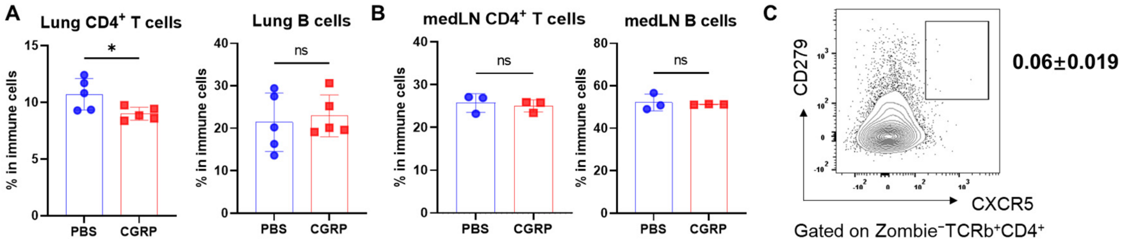

Figure S11. Frequencies of CD4$^+$ and B cells in lungs and medLNs, and detection of lung Tfh cells.

Adult mice were intranasally administered with HDM to induce allergic asthma. **(A–B)** Frequencies of CD4$^+$ T cells and B cells in the lung and medLNs following PBS or CGRP treatment, n = 4–5/group. Graphs depict individual values and group means ± SD. Statistical significance was determined using two–tailed Student's *t* test: ns, not significant; *P < 0.05. **(C)** Flow cytometry plot showing Tfh cells in lung, with quantification presented as mean ± SD, n = 4.

Table S1. Key resources table of antibodies

Antibodies	Source	Identifier
Purified anti-mouse CD16/32 Antibody	BioLegend, USA	Cat# 101302
APC/Fire 750 anti-mouse CD11c Antibody	BioLegend, USA	Cat# 117352
BB515 Rat Anti-Mouse CD25	BD Biosciences, USA	Cat# 564424
Brilliant Violet 510 anti-mouse CD45 Antibody	BioLegend, USA	Cat# 103138
Brilliant Violet 605 anti-mouse/human CD44 Antibody	BioLegend, USA	Cat# 103047
APC/Fire 750 anti-mouse/human CD11b Antibody	BioLegend, USA	Cat# 101262
Alexa Fluor 488 anti-mouse/human CD45R/B220 Antibody	BioLegend, USA	Cat# 103225
Brilliant Violet 510 anti-mouse CD8a Antibody	BioLegend, USA	Cat# 100752
PE/Cyanine7 anti-mouse TCRβ chain Antibody	BioLegend, USA	Cat# 109222
Pacific Blue anti-mouse CD4 Antibody	BioLegend, USA	Cat# 100531
APC anti-mouse CD62L Antibody	BioLegend, USA	Cat# 104412
Pacific Blue anti-mouse CD90.2 (Thy-1.2) Antibody	BioLegend, USA	Cat# 140306
Mouse ST2/IL-33R Antibody	Mdbio, USA	Cat# 101001PE
Alexa Fluor 647 anti-mouse Ly6G Antibody	BioLegend, USA	Cat# 127610
Brilliant Violet 510 anti-mouse CD45.1 Antibody	BioLegend, USA	Cat# 110741
BV605 Rat Anti-Mouse Siglec-F	BD Biosciences, USA	Cat# 740388
APC/Fire 750 anti-mouse CD335 (NKp46) Antibody	BioLegend, USA	Cat# 137632
Brilliant Violet 510 anti-mouse CD45.1 Antibody	BioLegend, USA	Cat# 110741
Brilliant Violet 510 anti-mouse FcεRIα Antibody	BioLegend, USA	Cat# 134327
BUV395 Rat Anti-Mouse Ly-6G and Ly-6C	BD Biosciences, USA	Cat# 563849
PE/Cyanine7 anti-mouse CD45.2 Antibody	BioLegend, USA	Cat# 109830
Brilliant Violet 605 anti-T-bet Antibody	BioLegend, USA	Cat# 644817
Alexa Fluor 647 anti-mouse CD69	BioLegend, USA	Cat# 104518
Gata-3 Monoclonal Antibody (TWAJ) Alexa Fluor 488	eBioscience, USA	Cat# 53-9966-42
Human/Mouse ROR gamma t/RORC2 Alexa Fluor 488 MAb (1181a)	BioLegend, USA	Cat# IC9125G-025

PerCP/Cyanine5.5 anti-mouse CD19 Antibody	BioLegend, USA	Cat# 152406
PerCP/Cyanine5.5 anti-mouse CD80 Antibody	BioLegend, USA	Cat# 104738
Brilliant Violet 605 anti-mouse CD86	BioLegend, USA	Cat# 105037
Pacific Blue anti-mouse I-A/I-E	BioLegend, USA	Cat# 107620
PE anti-mouse CD185 (CXCR5) Antibody	BioLegend, USA	Cat# 145504
Brilliant Violet 605 anti-mouse CD279 (PD-1)	BioLegend, USA	Cat# 135220
Brilliant Violet 605 anti-mouse Ki-67 Antibody	BioLegend, USA	Cat# 652413
PE anti-mouse IgE	BioLegend, USA	Cat# 406908
IL-4 Monoclonal Antibody (11B11), PerCP-eFluor 710	eBioscience, USA	Cat# 46-7041-80
IL-13 Monoclonal Antibody (eBio13A), eFluor™ 660	eBioscience, USA	Cat# 50-7133-82
CD95 (APO-1/Fas) Monoclonal Antibody (15A7) APC	eBioscience, USA	Cat# 17-0951-82
PerCP-Cy5.5 Rat Anti-Mouse CD38	BD Biosciences, USA	Cat# 562770

Table S2. Primer sequences for quantitative PCR

Name	Sequence (5’→3’)
Calcrl-F	CGGATGGCTATGCTGGAATGAC
Calcrl-R	GGATGCCGAAACCAGTGTCCAT
Ramp1-F	TGGTCTTTCCCCAGTCACAC
Ramp1-R	GGACCCTGACTATGGGACTCT
Actb-F	CATTGCTGACAGGATGCAGAAGG
Actb-R	TGCTGGAAGGTGGACAGTGAGG
